# Health outcomes of online consumer health information: A systematic mixed studies review with framework synthesis

**DOI:** 10.1002/asi.24178

**Published:** 2019-01-30

**Authors:** Pierre Pluye, Reem El Sherif, Vera Granikov, Quan Nha Hong, Isabelle Vedel, Maria Cristiane Barbosa Galvao, Francesca E.Y. Frati, Sophie Desroches, Carol Repchinsky, Benoît Rihoux, France Légaré, Bernard Burnand, Mathieu Bujold, Roland Grad

**Affiliations:** ^1^ Department of Family Medicine McGill University 5858, Chemin de la Côte‐des‐Neiges, Suite 300, Montréal QC H3S 1Z1 Canada; ^2^ Department of Social Medicine, Faculty of Medicine of Ribeirao Preto University of Sao Paulo Avenida Bandeirantes 3900, Ribeirão Preto, São Paulo CEP 14049‐900 Brazil; ^3^ Schulich Library of Physical Sciences, Life Sciences, and Engineering McGill University 809 Sherbrooke Street West, Montreal QC H3A 0C1 Canada; ^4^ School of Nutrition, Faculty of Agriculture and Food Sciences Laval University 2325, Rue de l'Université, Québec QC G1V 0A6 Canada; ^5^ Canadian Pharmacists Association 1785 Alta Vista Drive, Ottawa ON K1G 3Y6 Canada; ^6^ Centre de Science Politique et de Politique Comparée Université Catholique de Louvain Place Montesquieu 1/L2.08.07, Louvain‐la‐Neuve 1348 Belgium; ^7^ Department of Family Medicine and Emergency Medicine Laval University 1050, Avenue de la Médecine, Québec QC G1V 0A6 Canada; ^8^ Institut Universitaire de Médecine Sociale et Préventive Lausanne University Hospital Route de la Corniche 10, Lausanne CH‐1010 Switzerland

## Abstract

The Internet has become the first source of consumer health information. Most theoretical and empirical studies are centered on information needs and seeking, rather than on information outcomes. This review's purpose is to explore and explain health outcomes of Online Consumer Health Information (OCHI) in primary care. A participatory systematic mixed studies review with a framework synthesis was undertaken. Starting from an initial conceptual framework, our specific objectives were to (a) identify types of OCHI outcomes in primary care, (b) identify factors associated with these outcomes, and (c) integrate these factors and outcomes into a comprehensive revised framework combining an information theory and a psychosocial theory of behavior. The results of 65 included studies were synthesized using a qualitative thematic data analysis. The themes derived from the literature underwent a harmonization process that produced a comprehensive typology of OCHI outcomes. The revised conceptual framework specifies four individual and one organizational level of OCHI outcomes, while including factors such as consumers' information needs and four interdependent contextual factors. It contributes to theoretical knowledge about OCHI health outcomes, and informs future research, information assessment methods, and tools to help consumers find and use health information.

## Introduction

The increased access to the Internet and expectations for consumers to be more involved in healthcare decision‐making processes have resulted in an unprecedented demand for Online Consumer Health Information (OCHI; Anderson & Klemm, 2008). Consumer health information is “any information that enables individuals to understand their health and make health‐related decisions for themselves and their families” (Patrick, Koss, Deering, & Harris, 1995, p. 261). In contrast to personalized recommendations for specific conditions and patients (for example, algorithm‐based clinical decision support rule for treating a patient and that accounts for individual demographic and medical characteristics), OCHI consists of generic information for the general public (information consumers) including patients. The availability of OCHI satisfies the *Right to Know*, a fundamental right to functioning in democracies (Florini, 2007). Specifically, OCHI may improve the (a) empowerment of people regarding their health and self‐care; (b) engagement of patients in healthcare, for example, in treatment decision‐making; and (c) health outcomes (Baker et al., 2007; Edwards, Davies, & Edwards, 2009; Erdem & Harrison‐Walker, 2006; Smith & Duman, 2009; Suziedelyte, 2012).

Although numerous studies have examined Internet access, information needs, and retrieval, few have examined outcomes of information, in particular ultimate outcomes such as health outcomes; for example, three recent literature reviews reported only a few studies focusing on information outcomes (Case & Given, 2016; Case & O'Connor, 2016; Urquhart & Turner, 2016). Outside our work, no conceptual framework seems to currently link health outcomes of OCHI to information use, needs, and outcome‐related factors. Specifically, in community‐based primary healthcare (hereafter primary care), better understanding of OCHI health outcomes and associated factors is important for increasing positive outcomes and preventing negative ones. In primary care, there are numerous theoretical and empirical studies and literature reviews on the quality of information sources, patients' information needs, and information‐seeking behavior, but rare empirical studies focus on OCHI health outcomes in a comprehensive manner (Pluye, Grad, & Barlow, 2017). Existing primary care studies provide sparse evidence, but no studies yet have integrated knowledge on health outcomes of OCHI. Stated otherwise, this literature has not been reviewed systematically, nor is there any systematic review‐based theory on these outcomes and associated factors.

Therefore, the purpose of this review is to explore and explain health outcomes of OCHI in primary care and associated factors (theory‐generative research synthesis). Better understanding of these outcomes can assist information providers in assessing the value of information content retrieved from or delivered by information technology (how such content is valuable from the information consumers' viewpoint), and for managing information systems that take into account users' evaluation of the information. Knowledge of OCHI outcomes and associated factors can be applied in research and development of patient information aids, which can help patients to find relevant understandable information, and use it. Although there are aids for specific decision‐making situations (for example, clinical rules), there is no comprehensive generic aid for helping patients to find and use appropriate OCHI for all kinds of health situations.

This help can be significant. In 2015, about 90% of the population was connected to the Internet in the United States, which is now the most frequently accessed platform for finding consumer health information (Anderson & Perrin, 2016). The numbers of Internet users are similar in countries of the Organization for Economic Co‐operation and Development (OECD); for example, nearly 87% of Canadian households were connected to the Internet in 2013 (Canadian Internet Registration Authority, 2015). The use of information from the Internet is not limited to younger people; for example, 47% of the Quebec population aged 55 and older regularly uses the Internet, and this proportion is increasing rapidly (CEFRIO, 2011). The most frequent activity on the Internet after email is searching for OCHI (Powell, Inglis, Ronnie, & Large, 2011).

Our general research question is as follows. For primary care information consumers (including patients), what are the key health outcomes of OCHI and associated factors? In the first section of this article, we define the main concepts and present an initial conceptual framework of information outcomes. Subsequently, we report a systematic literature review of qualitative and quantitative evidence that led to the revision and improvement of this framework. We conclude with a discussion of lessons learned from our results, contributing to future research and development of patient information aids.

## Conceptual Boundaries

Primary care encompasses health and social services, as well as disease prevention, health promotion, and population health functions (Muldoon, Hogg, & Levitt, 2006; Starfield, Shi, & Macinko, 2005; World Health Organization, 2014). It is usually the first point of contact with the healthcare system. It aims to support individuals and families to make the best lifelong decisions for their health (continuity of care). In primary care, patients can play active roles, and frequently search for health information (Frenk, 2009). As patient‐centered services increase, information providers are producing more user‐centered online resources (Smith & Duman, 2009). Although some experimental studies measured outcomes of OCHI in oncology and public health, findings from these studies are not necessarily transferable to primary care. For example, randomized controlled trials demonstrate that OCHI can contribute to reduce depression in cancer patients, 20% of whom are affected by depression in reaction to or aggravated by cancer (D'Souza, 2013). However, this finding may not translate to primary care where depression is not necessarily due to cancer or any other chronic disease. In primary care, information needs pertain to a large diversity of areas and topics.

**Figure 1:**
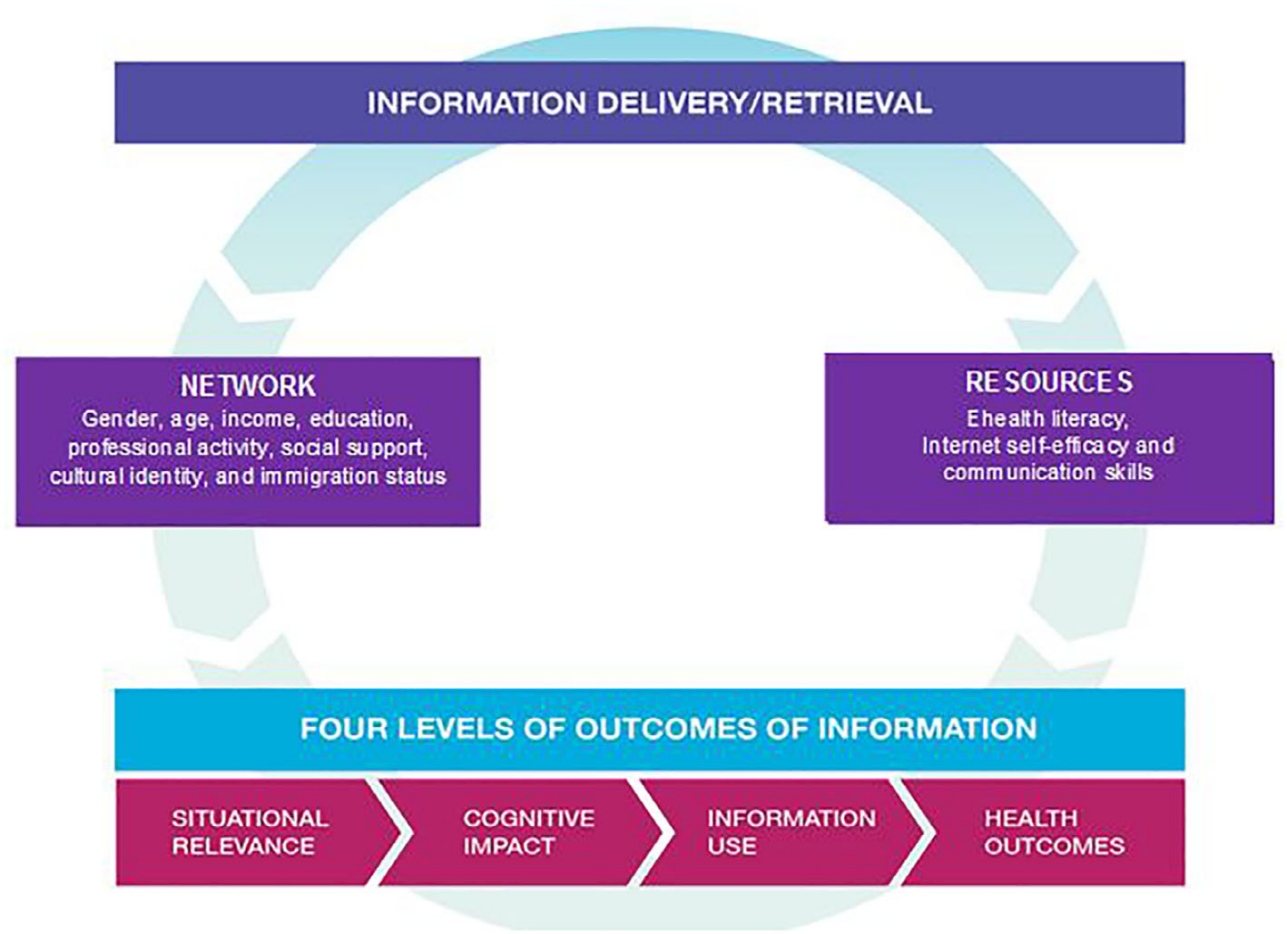
Initial conceptual framework: Four levels of outcomes of information. [Color figure can be viewed at wileyonlinelibrary.com]

Our systematic review is based on an initial conceptual framework, represented in Figure 1. This framework needs revision and improvement. It has been developed in an iterative manner using information studies and theories, and qualitative research with online health information consumers. It is consistent with contemporary multifaceted approaches to human information behavior, which combine cognitive approaches (for example, psychological and behavioral factors) and social approaches (for example, affective and contextual factors; Pettigrew, Fidel, & Bruce, 2001). The framework is described in the following six paragraphs: two on OCHI outcomes and four on outcome‐related factors. The first two paragraphs summarize the information theory and the 16‐year research journey (citing 3 of 62 OCHI outcome‐related publications) that led us to propose four levels of outcome of information. The next four paragraphs summarize about 30 other information studies and reviews that influenced the conceptualization of the outcome‐related factors.

Recent reviews showed few studies on information outcomes such as a change in knowledge, emotion, intention, or behavior after information is found or received; they presented exemplar studies, but did not identify types of outcomes in a comprehensive manner (Case & O'Connor, 2016; Urquhart & Turner, 2016). Since 2001, we have progressively developed levels of outcomes included in the initial framework using the “Value of information” overarching construct and the “Acquisition Cognition Application” model proposed by Saracevic and Kantor (1997). The four levels of outcomes of information delivery and retrieval are: situational relevance, cognitive/affective impact, use, and subsequent health/well‐being outcomes of information (Pluye et al., 2013, 2014). These levels reflect the value of information (how information is valuable) from the consumers' viewpoint and are derived from an iterative three‐stage process: people receive or retrieve information (acquisition), may understand and integrate it (cognition), and possibly use it (application). For example, relevance of information is the first and foremost value of information, and can be seen as a measure (hereby an outcome) of relevance behavior, which is defined as a dynamic iterative selection process using a wide range of clues to screen and retain information content preceding in‐depth cognition (Saracevic, 2007a, 2007b). For each level, a series of literature reviews and empirical studies led us to identify different types of outcomes (Bujold et al., 2018; Pluye et al., 2014).

By way of illustration, a primary care patient accesses (acquisition) a particular webpage (information object) to answer a personal health question before an encounter with a professional (a specific situation). Level‐one: the information answers her/his question (situational relevance of information). Level‐two: s/he understands the information and learns something new about healthcare (cognitive impact of information). Level‐three: s/he applies the information to modify his/her health management plan and discuss it with a professional (information use). There are four main types of information use: conceptual, legitimating (a plan/action), instrumental and symbolic (these two being illustrated here). Level‐four: due to this information use, her/his worries decrease (health outcome).

With respect to outcome‐related factors, the initial framework was patient‐centered and contextual; that is, included the social context, interaction, and discourse through which the sharing of information occurs (Savolainen, 2002). In this framework, patients were deemed active interpreters (reflexive patients) of OCHI in specific situations (Dervin, 2003). Although information studies traditionally focus on working environments and workers, for example, clinical settings and health professionals (Savolainen, 2007), the initial framework focused, instead, on information use in everyday life. Information theories successively defined the notion of context as the social environment (Wilson, 1999, 2006), the community and culture, the knowledge and power system, and the social environment temporarily created when people share information (Fisher, Landry, & Naumer, 2006). In accordance with Courtright's literature review (2007), two key contextual elements influence information use: the patient's network and resources.

The patient's network can be determined by gender, age, income, education, professional activity, cultural identity, immigration status, and social network (among other factors), which are common factors of information seeking and use. With respect to patient resources, three main individual factors influence an individual's search for and use of information: e‐health literacy, Internet self‐efficacy, and communication skills. Together, these factors determine the extent to which information is accessed and how it is used by patients. The first factor, e‐health literacy, integrates computer literacy, information literacy, and health literacy, which are interdependent (for example, a person with a low literacy level also has a low level of health literacy). Literacy level is generally defined as the degree to which a person has the ability to acquire, understand, evaluate, and use information needed to obtain services and make appropriate decisions (Kindig, Panzer, & Nielsen‐Bohlman, 2004; Ronson McNichol & Rootman, 2016). Direct acquisition of information depends on a person's ability to find information adapted to their individual literacy level (van Deursen & van Dijk, 2015; Yu, 2006; Zach, Dalrymple, Rogers, & Williver‐Farr, 2012). A low level of health literacy is defined as a difficulty in acquiring, understanding, and applying health information by oneself (Murray, Hagey, Willms, Shillington, & Desjardins, 2008). In this framework, literacy level is both individual and contextual, given that a social network can compensate for an individual's low literacy level. Literacy level is also situational, as sickness‐related emotion and stress may momentarily lower an individual's health literacy level (Nahl & Bilal, 2007). In addition, culture is central in health literacy, as a literacy level depends on a person's ability to understand systems of symbols from the dominant culture and language supporting health information (Ronson McNichol & Rootman, 2016).

The second factor, Internet self‐efficacy, is one's belief in one's ability to complete online tasks. People with higher levels of self‐efficacy are more comfortable using the Internet to seek consumer health information and use it for decision‐making (Xie & Bugg, 2009). Self‐efficacy and information‐seeking behavior vary along a continuum from inquisitive and autonomous people who look for all kinds of information by curiosity, to people who selectively choose only the most pertinent information when needed, to people who may avoid some types of information (Case & Given, 2016; Loiselle, Lambert, & Dubois, 2006; Santana et al., 2011). A low level of Internet self‐efficacy alone does not prevent information use and outcomes. Even the homeless, recent immigrants, and refugees can acquire information on the Internet directly (high level of Internet self‐efficacy) or mediated by their social network (low level of Internet self‐efficacy): their networks include community organizations, public libraries, and education, health, and social services (Britz, 2004; Chatman, 1996; Hersberger, 2003; Knapp, Madden, Wang, Sloyer, & Shenkman, 2011; Lloyd, Kennan, Thompson, & Qayyum, 2013; Ronson McNichol & Rootman, 2016).

The third factor is communication skills. Good communication skills may allow people to overcome low levels of e‐health literacy and Internet self‐efficacy (Zach et al., 2012). Mediated (by someone else) acquisition of information is very common; for example, up to 18.1% of searches for parenting information may be conducted for someone else's child and communicated to the parents (Pluye et al., 2015). Good communication with health professionals usually leads to better health outcomes (Street, 2013). Consumers combine professionals' information (mediated acquisition) with direct acquisition of information, allowing them to further probe information provided by professionals such as health practitioners, librarians, and social workers. We conceive of these combinations as the interpenetration of social systems centered on communicative action; for example, the health system (mediated access), and a consumer system (direct access and mediated access via their social network; Luhmann, 1992, 1995, 1999).

However, this initial framework faced at least four limitations. First, it was patient‐centered (not consumer‐oriented). Second, it was composed of 30 factors and outcomes grouped in seven themes (Figure 1) that were derived from clinical studies, few interviews (no systematic review of OCHI studies) and five potential overarching information theories (Dervin, 2003; Fisher et al., 2006; Saracevic & Kantor, 1997; Savolainen, 2002, 2007; Wilson, 1999, 2006). Third, it included only positive individual patient health outcomes (no negative health outcomes). Fourth, although there is a continuum between cognitive impacts and conceptual or legitimating use of information, there are a few steps between cognitive impacts and instrumental or symbolic use of information (no behavioral theory).

The initial framework was a proof‐of‐concept that needed to be revised and improved using a systematic literature review on OCHI. In accordance with a definition of types of theories (Gregor, 2006), our general goal was to develop a “theory for explaining,” that is, analyze, describe, and explain health outcomes of OCHI in a primary care context. Starting from an initial conceptual framework, our specific objectives were threefold: (a) to systematically identify types of OCHI outcomes according to primary care research studies, (b) to identify factors associated with these outcomes, and (c) to integrate these factors and outcomes into a comprehensive conceptual framework combining an information theory and a psychosocial theory of behavior.

## Methodology and Methods

We conducted a participatory systematic mixed studies review with framework synthesis (Carroll, Booth, Leaviss, & Rick, 2013). The rationale for choosing this methodology was that we started from an initial framework (derived from information studies and a few interviews), which needed to be revised and improved using a systematic literature review of studies on OCHI in primary care. Mixed studies reviews and corresponding types of synthesis are presented in Appendix 1 to justify our use of a framework synthesis (compared to other types of syntheses), and to help readers who are not familiar with these methodologies and methods.

This review is reported in accordance with the PRISMA statement for quantitative systematic literature reviews (reviews of quantitative studies with meta‐analysis of quantitative evidence; Liberati et al., 2009), and the ENTREQ statement for enhancing transparency in reporting a qualitative review (literature review of qualitative research studies with synthesis of qualitative evidence; Tong, Flemming, McInnes, Oliver, & Craig, 2012). No specific standard for reporting mixed studies reviews was found in a reference source on guidelines for reporting research (www.equator-network.org).

### *Approach*

Our team includes researchers, graduate students, primary care professionals, and information specialists. This review was undertaken using an organizational participatory approach with a professional organization partner (Canadian Pharmacists Association) who provided input at the planning stage (knowledge gaps, research needs, review question, and objectives) and throughout. Contacts were more frequent at the start of the review, during the data collection phase, by means of a blog where we posted updates, and invited feedback via a “Comments” section. To obtain input for drafting the inclusion/exclusion criteria, a quick survey was created that allowed all team members (including our partner) to flag which criteria were in need of clarification. The results of each step were circulated to all team members, and their feedback was incorporated.

### *Design*

We conducted a systematic review of qualitative and quantitative evidence, known as systematic mixed studies review (Hong, Pluye, Bujold, & Wassef, 2017; Pluye & Hong, 2014; Pluye, Hong, Bush, & Vedel, 2016). This emerging form of literature review applies mixed methods research in the field of literature reviews and provides a rich and highly practical understanding of complex health interventions (Grant & Booth, 2009; Heyvaert, Hannes, & Onghena, 2016; Pluye, Gagnon, Griffiths, & Johnson‐Lafleur, 2009). Main features of mixed studies reviews are presented in Appendix 1.

### *Eligibility Criteria*

The following inclusion criteria were used: studies pertaining to the use of OCHI, or health outcomes of OCHI, or both; empirical studies on primary care; and studies focusing on general information about health and medical topics. An empirical research study was defined as an original qualitative, quantitative, or mixed methods study. Studies on primary care pertained to (a) primary care topics such as health promotion, disease prevention, early detection, diagnosis, and treatment of disease; (b) primary care social actors, specifically the general public, patients and health practitioners such as community pharmacists, family physicians, and nurse practitioners; and (c) primary care services, namely, first‐contact care, care coordination, care over time, and comprehensive care in community‐based healthcare organizations. Stated otherwise, all primary care studies (all health problems) were included, whereas studies concerning hospitals and hospitalized patients with specific diseases or types of disease (for example, cancer) were excluded. General information was distinguished from clinical decision support systems, which refer to algorithm‐based clinical rules (calculators) using specific individual data (Simon, 1980).

### *Information Sources*

Nine bibliographic databases were searched: Medline (Ovid), Embase (Ovid), PsycINFO (Ovid), CINAHL (Ebsco), LISA (ProQuest), ERIC (ProQuest), Cochrane Library (Wiley), Library, Information Science & Technology Abstracts (Ebsco), and British Nursing Index (ProQuest). In addition, lists of references in literature review articles and key textbooks about OCHI were scrutinized. The gray literature was also searched using Google Scholar because not all evaluation studies on programs about information are necessarily published and indexed in bibliographic databases. After the selection stage, additional potentially relevant records were retrieved by tracking the citations (snowballing) of the selected documents. Both the reference lists of these documents (articles cited), and the publications citing these documents, were independently reviewed by two reviewers. This citation tracking was conducted using the ISI Web of Science and Scopus databases, and continued up to saturation (no additional pertinent studies found).

### *Search Strategy*

The bibliographic database search strategies were developed by four specialized librarians. The search covered studies published from 1990 (launch year of the World Wide Web) to July 2014 with no language restrictions. The search strategy was developed for Medline (Ovid) and adapted for other bibliographic databases (Appendix 2). A mixed filter that combines five filters for retrieving publications reporting empirical studies (randomized controlled trials, nonrandomized studies, descriptive quantitative studies, qualitative research, and mixed methods research) was used. This filter has been tested and found to have high recall (El Sherif, Reem, Pluye, Gore, Granikov, & Hong, 2016). The gray literature search was conducted by a specialized librarian with OCHI expertise.

### *Selection of Relevant Studies*

Records (authors, title, source, year, abstract, keywords) were imported into EndNote X7, and duplicates removed (Bramer, Giustini, de Jonge, Holland, & Bekhuis, 2016). Records were then imported into specialized online software for coding (DistillerSR). For each record, two reviewers independently assigned eligibility codes. When reviewers agreed that records were potentially relevant, the corresponding full‐text publications were sought. Records were excluded when reviewers agreed that they were irrelevant. Corresponding full‐text publications were also sought when reviewers disagreed about the relevance of the records. Again, full‐texts were imported into DistillerSR and coded using eligibility criteria. Discrepancies between reviewers' responses were usually resolved by discussion. Those that were not resolved easily were referred to a third party (Higgins & Green, 2011).

### *Quality Appraisal of Included Studies*

Because studies with diverse methods (qualitative, quantitative, and mixed) were included, the methodological quality was assessed using the Mixed Methods Appraisal Tool (MMAT; Pluye et al., 2009). The MMAT is a validated tool and has been tested for reliability (Crowe & Sheppard, 2011). Two reviewers independently assessed the included studies using the 2011 version of the MMAT (Pace et al., 2012; Souto et al., 2015). Any discrepancy between reviewers' appraisal was usually resolved by discussion. Disagreements that were not resolved easily were referred to a third party. No studies were excluded based on the appraisal. The appraisal contributed to the description of the characteristics of included studies (Appendix 3).

### *Data Extraction and Synthesis of Included Studies*

In line with guidance for qualitative research, a theory‐based transparent and reproducible synthesis was conducted (Srker, Xiao, & Beaulieu, 2013). Specifically, results of included studies were extracted and analyzed using a “best fit” framework synthesis method, the objective of which is to revise an existing framework (Carroll et al., 2013). The synthesis consisted of coding qualitative and quantitative evidence (extracted data) against the initial framework (Figure 1), and producing a revised framework combining two commonly cited theories: one from information studies and one from psychosocial behavioral research, a combination deemed fruitful (Greyson & Johnson, 2016). The former theory focuses on information behavior, and informed our overall “information context, needs, behavior, and outcome” frame (Wilson, 1981, 1999). Among the five main information theories that influenced our work, Wilson's theory refers to an overarching generic “macro‐behavior” model (cited more than 2,000 times according to Scopus, thereby well‐tested and supported) including “feed‐back loops” that were integrated into our revised framework (Wilson, 1999, p. 257).

The latter focuses on knowledge, attitude, and behavior, which informed the outcome‐related subthemes, specifically the steps between cognitive impacts and information use (Fishbein & Ajzen, 2011). In health behavior research and psychology, the Theory of Reasoned Action is generic, and often called the KAB theory (Knowledge Attitude Behavior; Glanz, Rimer, & Viswanath, 2008; Godin, 2012). Indeed, “various theoretical models of health behavior reflect the same general ideas” (Glanz et al., 2008, p. 28); for example, the Health Belief Model, the Social Cognitive Theory, the Transtheoretical Theory, and recently the Behavior Change Wheel (Bandura, 2004; Michie, Van Stralen, & West, 2011; Prochaska, Redding, & Evers, 2008; Rosenstock, Strecher, & Becker, 1988). For all these theories, the literature suggests numerous new variants, benefits, and pitfalls. Yet it is difficult to say which one is the best (Glanz et al., 2008; Godin, 2012). Therefore, we chose the KAB model, as it fits well with our levels of outcomes, and is generic and cited more than 50,000 times (Fishbein & Ajzen, 2011), which shows that a large body of research tested and supported it. This is a criterion for choosing one among “the proliferation of health behavior theories” (Glanz et al., 2008, p. 28).

We followed the following four phases.

**Phase 1. Coding data and creating new themes.** A hybrid thematic synthesis (deductive/inductive) was performed (Fereday & Muir‐Cochrane, 2006). We used key elements of our initial framework (Figure 1), specifically the four levels of outcomes, and “network” and “resources” (influencing factors) as starting themes to synthesize the data. Concomitantly, we identified new themes suggested by the data. Using specialized software (NVivo 10), all included studies were shared among three reviewers with experience or training in qualitative data analysis. For each study, two reviewers independently coded the text (assigned extracts of text to themes), and created new themes as needed. In addition, each reviewer kept a detailed research diary to explain their coding and coding‐related difficulties, new themes, and justification for their creation. The third reviewer discussed the coding with the two other reviewers, and noted his comments in a research diary.

In line with traditional guidance for consistency and rigor in qualitative thematic data analysis (Boyatzis, 1998) and its application as “qualitative thematic synthesis” in literature reviews (Thomas & Harden, 2008), our synthesis was based on an interpretative method and research meetings, where coding processes and diaries were shared and discussed. A meeting was held among the three reviewers following the preliminary analysis of the first 10 studies. All new themes, corresponding data, and reviewers' concerns were discussed until reviewers agreed on themes, their definitions, and a few illustrative examples (data‐based quotes). Twice per month, meetings were held until all studies were coded and analyzed. Then a within‐study and cross‐study analysis was conducted to check whether the new themes (suggested by data) added on old themes (from the initial framework). After further discussions, themes were kept or refined for the synthesis, or removed from the next steps (for example, interesting theme, but not applicable to consumers). Phase 1 led to identifying themes pertaining to OCHI outcomes, information needs driving seeking behavior, and contextual factors.

**Phase 2. Harmonizing themes pertaining to OCHI outcomes.** With regard to our first objective, a harmonization process was achieved for themes pertaining to OCHI outcomes. This led to further refining and clarifying results of the Phase 1 thematic analysis. In line with information studies, harmonizing themes consist of clearly defining them to produce a terminology including of key terms, concept definitions, and examples (International Organization for Standardization, 2007). Key terms are words, compound words, or multiword expressions that in specific contexts are given specific meanings. Concept definitions precisely define key terms (Pavel & Nolet, 2002). The harmonization was conducted by three researchers, including an information scientist with expertise in terminologies. For each outcome‐related theme, we identified terms that designate outcomes and confirmed the usage of these terms and their concepts in reference documents such as dictionaries. Then the terms were organized considering their superordination, subordination, coordination, and equivalence relationships. When necessary, we improved the terms through lexical additions to better reflect the underlying specific concept (avoiding ambiguities and conceptual superimposition). For each level of information outcome, we created a more accurate terminology, grouped, and clarified types of outcomes. The first version of this terminology was reviewed, discussed, and refined by all research partners, which created a second version (hereafter the typology of OCHI outcomes).

**Phase 3. Grouping themes pertaining to factors associated with OCHI outcomes.** With regard to our second objective, we identified contextual factors and OCHI needs driving seeking behavior. Using the harmonization process, themes pertaining to the latter, derived from Phase 1 thematic analysis, were further refined and clarified. Such a harmonization process was not deemed necessary for contextual factors, which were simple (for example, age and gender).

The themes corresponding to contextual factors influencing OCHI needs and outcomes were categorized and grouped using a card‐sorting exercise (Rugg & McGeorge, 2005). To this end, each theme was written on one side of a large cue‐card with a corresponding key data‐based excerpt on the other side. Six members of the research team met to examine each card and compare it to others. Some cards (themes) were deemed similar and grouped into a pile, whereas others were viewed as too broad and divided into subthemes for which new cue‐cards were made. Using an iterative process, all cards (themes) were categorized and grouped until team members reached consensus. This led to four major categories of themes (four sets of cards). To facilitate this collaborative analytical process, the meeting was audiorecorded, photographs of key moments were taken, and one team member took notes.

**Phase 4. Producing a revised framework.** With regard to our third objective, our initial framework was revised through all previous phases. In this last phase, we adopted an iterative collaborative process over a series of meetings. In the first meeting, three members of the research team placed all major themes into text‐boxes and added these boxes to the figure representing the initial framework (F1), which created a second version of the framework (F2). Over the course of the next six meetings, they proposed different variants of F2, discussed them, and modified the text‐boxes and the relationships between them; thus, proposed alternative figures. Concomitantly, they also examined literature reviews of the main theories of information‐seeking behavior, information use, behavioral, psychological, and sociological research. As mentioned, they incorporated two theories that modified the boxes and figures (Fishbein & Ajzen, 2011; Wilson, 1981, 1999) and complemented the two theories used in the initial framework (Leonardi, 2011; Luhmann, 1995). Consensus on a “best fit” framework was reached at the sixth meeting, and a third version of the framework (F3) was produced. This was reviewed by all team members. Their feedback comments led to a fourth version (F4), which was presented at two international research meetings where feedback from other researchers was gathered and incorporated into the current framework (F5).

## Results

### *Included Studies*

**Figure 2:**
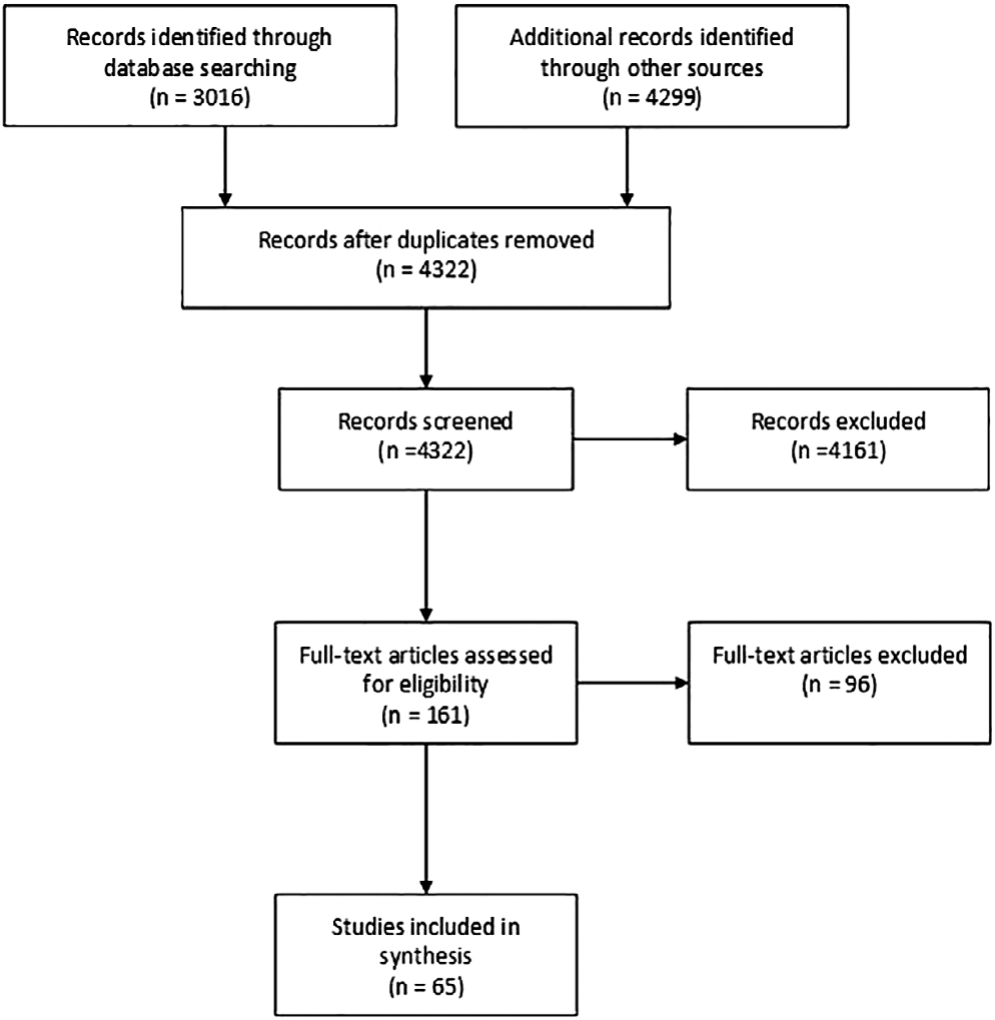
Flow diagram.

Of 4,322 unique records identified in our search, 65 studies that fulfilled our eligibility criteria were included in the review (Figure 2). There were 51 quantitative studies, 11 qualitative studies, and three mixed methods studies.

### *Study Characteristics*

The included studies are described in Appendix 3. Study participants were either consumers of online health information (including patients, family caregivers, and the general public), or family physicians reporting outcomes derived from patients using OCHI (outcomes on clinician–patient relationships and health services). Five of the included studies examined a specific intervention such as “information prescription” (Coberly et al., 2010) and “information retrieval training” (Campbell, 2009). Included studies were conducted in 15 countries (number of studies): Australia (*n =* 2), Australia and New Zealand (*n =* 1), Canada (*n =* 4), China (*n =* 1), France (*n =* 1), Israel (*n =* 2), Italy (*n =* 2), Japan (*n =* 1), Korea (*n =* 1), Pakistan (*n =* 1), Poland (*n =* 1), Switzerland (*n =* 3), Turkey (*n =* 1), UK (*n =* 12), and USA (*n =* 32). Based on what was reported in publications, 40 included studies can be considered of higher methodological quality (scoring 75% or 100% with the MMAT), whereas 25 can be seen of lower methodological quality (scoring 25% or 50% with the MMAT). The 65 studies were included in the synthesis.

### *Results of the Synthesis*

#### *Harmonization of levels of outcomes*

With regard to our first objective, the harmonization process led to producing a typology of OCHI outcomes in primary care. Specifically, these results cast light on ultimate outcomes (health outcomes and outcomes affecting healthcare services) and linkages to intermediary outcomes (such as cognitive impact and use) of information. A typology including 19 key terms with concept definitions and examples from included studies is presented in Appendix 4. For each key term, subterms were defined with examples from included studies. The typology includes the four initial individual levels of outcomes (key terms: situational relevance, cognitive impact, use, and health and healthcare‐related outcomes) and a fifth level of outcomes, which was uncovered through the data synthesis process (new key term: outcomes affecting healthcare services). This level is new (not in the initial framework) and includes two types of outcomes. First, it refers to a change in a healthcare service following consumers' behavioral information use, resulting in an increase or a decrease in the utilization of health services; for example, encourage or prevent a consultation with a health professional. Second, it refers to a change from the health professional's perspective; for example, a change in the professional's attitude and behavior during a clinical consultation when information is brought up by the patient.

#### *Groups of factors associated with OCHI outcomes*

**Figure 3:**
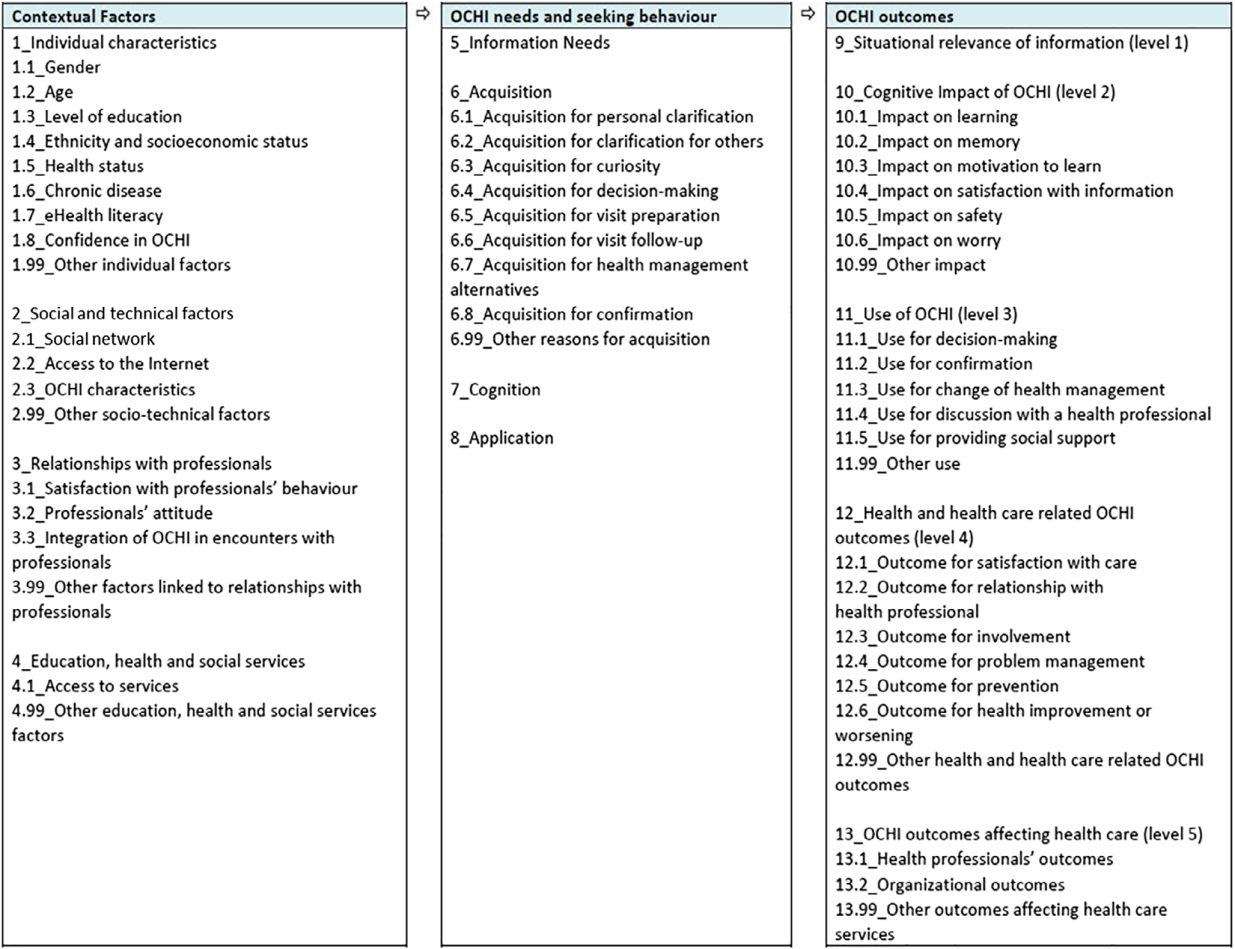
Contextual factors, OCHI needs, and outcomes.

With respect to our second objective, the list of contextual factors, OCHI needs and seeking behavior, and outcomes of OCHI, is presented in Figure 3. The harmonization process led to identifying eight types of consumers' reasons for acquiring online health information, which are presented in Appendix 5 with definitions and examples from included studies. They constitute a liaison between contextual factors and OCHI outcomes. They consist of reasons for the imbrication of a consumer in a specific situation with a particular OCHI. In addition, the card sorting exercise led to group the 15 themes pertaining to contextual factors into four main categories: individual characteristics, social and technical factors, patient–professional relationships, and education–health–social services. These categories and corresponding factors are presented in Appendix 6 with definitions and examples from included studies.

#### *Revised conceptual framework*

**Figure 4:**
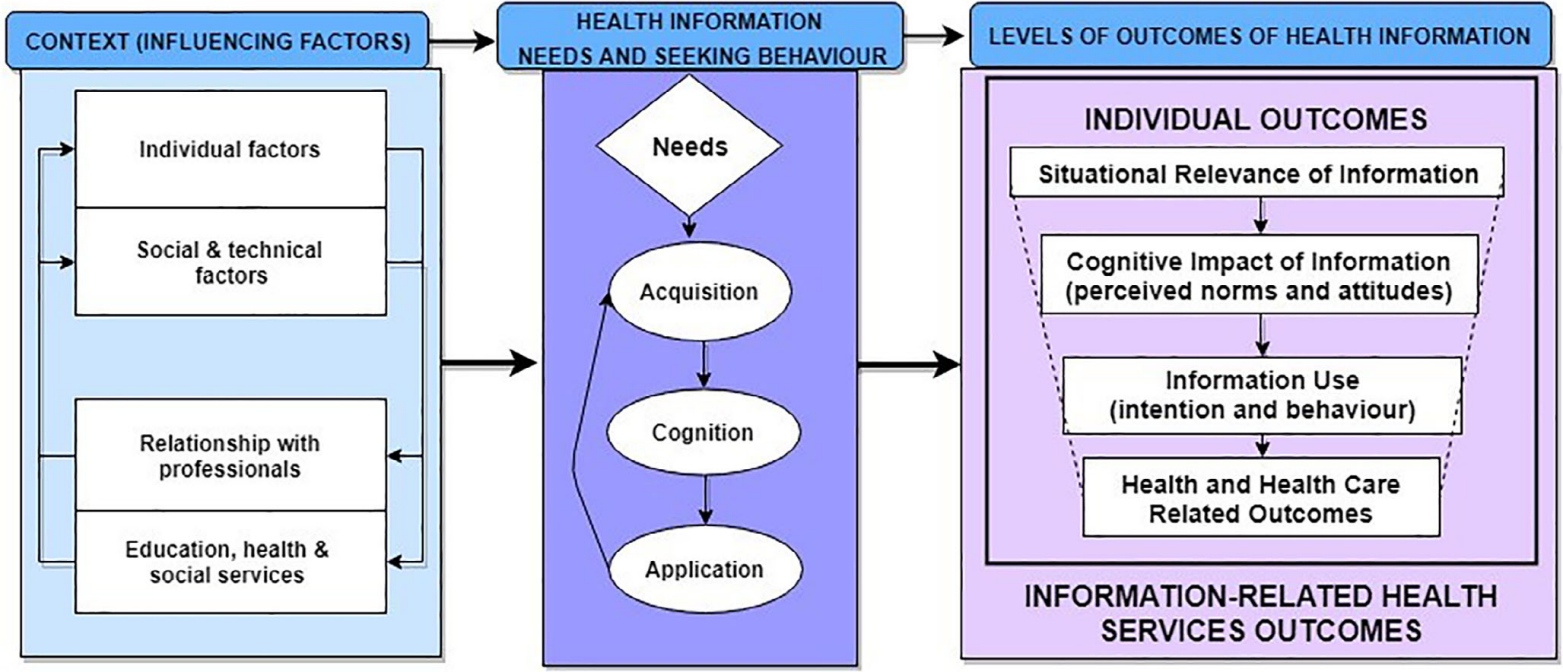
Revised framework: Health outcomes of online consumer health information.

Regarding our third objective, the revised conceptual framework is presented in Figure 4. The overarching construct is outcomes of OCHI. This includes three main interdependent subconstructs: the individual context (influencing factors) directly affects information needs and seeking behavior, which leads to five levels of outcomes of information. This framework is theoretical, as it provides an abstract explanation of OCHI in primary care (15 factors → 5 levels of outcomes), which can be tested using empirical research. Such a testing may suggest hypotheses in terms of likelihood and significance of the association between factors and outcomes, thereby transforming the framework into a predictive theory (Glanz et al., 2008). Although the application is limited to primary care, this area of application is quite broad, as everybody is involved in primary care at least once in their life. In line with Gregor (2006), the revised framework attempts to explain what information outcomes are, why‐how‐when‐where they occur, and for whom they occur (analysis and explanation being two types of theory), as shown in the following nine paragraphs.

***What are the OCHI outcomes?*** The revised framework includes four individual levels of OCHI outcomes (situational relevance, cognitive impact, and use of information, and health and healthcare outcomes of information) and one organizational level (information outcomes affecting healthcare services). First, the situational relevance of information constitutes the initial individual level of outcome; for example, people start reading a webpage and skip it when the title (or bottom line) seems irrelevant, while they read it when information is deemed relevant to their needs and situation.

Second, relevant information has a cognitive impact, which the user may perceive as positive or negative (for example, people might not understand the information content of a webpage), and consists of a change in a perceived norm or in an attitude (for example, people can learn something new). Relevant information with positive cognitive impact is not necessarily used as one can learn something new, but may not be in the situation where this new knowledge can be used; for example, people reported that because of the information found on the Internet, they wanted to look for more health information.

Third, studies reported types of information use; for example, people may use information to decide to consult health professionals. In addition, people may intend to use information, but actually use only a fraction of it (behavioral use). Relevant information with positive cognitive impact and use does not necessarily lead to health outcomes, as one can be reassured by the found information, use it in a conceptual or legitimating manner, and not expect any health change from using it.

Fourth, information use can contribute to individual health and well‐being outcomes; for example, our results suggest people might feel reassured (positive outcome) or more anxious (negative outcome) from using information. The level “health outcome” in the initial framework was replaced with “health and healthcare outcome” in the revised framework. The data suggested subthemes pertaining specifically to healthcare. For example, a subtheme “validation of the information with a health professional” corresponds to when patients felt more confident because the physician confirmed the trustworthiness of the information they presented during the clinical encounter. Based on our results, each type of “health and healthcare outcome” can be positive or negative, as seen in the revised framework (for example, “feeling pleased or not” for satisfaction with care).

Fifth, our results suggest an organizational level of OCHI outcomes, which was not in the initial framework. At this level, information use may lead to outcomes that affect the delivery of healthcare services; for example, people reported information‐related improvement in their ability to manage a health issue by themselves, which prevented them from consulting health professionals and decreased their utilization of healthcare services. This organizational level of outcomes is part of the revised framework. For each type of “outcome affecting healthcare services,” our results suggest positive and negative outcomes, which have been integrated in the revised framework. For example, “an increase or a decrease in health system use” can correspond to an appropriate use from a professional perspective, or an inappropriate use (overuse or underuse).

With regard to the first four levels, themes and subthemes were structured as pertaining to consumers' perceived norms, attitudes, intention to use information, and behavioral use in line with a Reasoned Action approach (Fishbein & Ajzen, 2011). Each level has been revised, and coherence has been improved to better reflect the consumers' perspective of information value (how information is valuable for them). Levels, themes, and subthemes are centered on consumers as decision‐makers in their own health and healthcare, which is coherent with a primary care context (Pluye et al., 2017). In addition, our results suggested three types of negative outcomes that have been integrated in the revised framework: “deterioration of the patient–physician relationship,” “negative patient health outcome,” and “time‐consumption or misuse of health services” (misuse combining underuse and overuse). For example, studies reported that OCHI may lead to unnecessary visits to a physician, which can take up more of their physician's time and leave less time for other patients.

***Why and how do these outcomes occur?*** The OCHI outcomes depend on patients' information needs, information‐seeking behavior (acquisition, cognition, and application of information) and contextual factors. The Phase 3 of the framework synthesis led us to incorporate consumers' information needs that drive seeking‐behavior and contextual factors into our revised framework, in line with Wilson's model of information‐seeking behavior (Wilson, 1981, 1999). In the revised framework, individual characteristics (for example, age) and social and technical factors (interrelated social and technical factors; for example, social support for finding, understanding, and using relevant information) are presented together because the latter (social network) can overcome personal low level of e‐health literacy. Moreover, the relationships with professionals (for example, integration of OCHI in encounters with professionals) are presented with education, health, and social services (for example, access to services) as they are interdependent factors. The primary care patient–professional relationship can influence information outcomes, which can in turn influence this relationship. In accordance with Luhmann's theory of social systems (Luhmann, 1992, 1995, 1999), consumers combine direct acquisition of OCHI with information from a health professional. This combination refers to the interpenetration of social systems centered on communicative action: the consumer system (direct access and mediated access via their social network) and the health system (mediated access).

***When and where do these outcomes occur?*** OCHI outcomes and associated factors are defined in relation to a specific information‐seeking situation: a particular information object is acquired or delivered (for example, a webpage or a newsletter) in a particular situation (for example, before or after a primary care patient–clinician encounter). These two conditions are necessary to observe the “imbrication” between information objects, technology, and users, who are the ultimate decision‐makers about the value of information (how the information is valuable for them; Leonardi, 2011). Imbrication represents the interdependency between people (human agencies) and information objects (material agencies) in a given situation. For example, “when they become imbricated (interlocked in particular sequences), they together produce, sustain, or change either routines or technologies” in a workplace (Leonardi, 2011, p. 149). On one hand, imbrication means that people have the capacity to form and achieve objectives; for example, answer health‐related questions using information such as a nutrition recommendation, then implement the retrieved recommendation. On the other hand, it means that information also acts somehow “on its own” and people do not entirely control the retrieval and delivery of these recommendations. Inspired by the structuration theory (social actors and structures influencing each other), imbrication reconciles two extreme worldviews: people do what they want (free will) versus they are constrained by their environment and state‐level policies (contingency). Imbrication is an underlying principle of the initial and revised frameworks, as it justifies why information may contribute to outcomes in specific situations, which cannot be replicated the same way over time, that is, imbrication at Time 1 influences the way it occurs at Time 2.

***For whom do these outcomes occur?*** Our conceptual framework has been developed for, and is centered on, primary care information consumers including patients. In accordance with the Canadian Institutes of Health Research (CIHR) Strategy for Patient Oriented Research (SPOR), we define patient as an overarching term that includes individuals with personal experience of a health issue and caregivers including family and friends. Stated otherwise, primary care patients are not necessarily people having symptoms and receiving medical care; they may have no symptoms, disease, or illness when they interact with information and report information outcomes. In the included studies, people reported information outcomes in primary care that (a) refers to a spectrum of health, education, and social services (first point of contact and continuity of care); (b) involves the coordination and provision of services (ranging from health promotion to prevention, diagnosis, and treatment of diseases) provided by dentists, dietitians, nurses, pharmacists, physicians, psychologists, public health practitioners, and social workers; and (c) is provided in a range of community settings including homes, clinics, physician offices, public health units, hospices, and the workplace.

## Discussion

This systematic review leads us to propose a typology of positive and negative health outcomes of OCHI in a primary care context. Our results contribute to knowledge via a comprehensive conceptual framework that includes factors associated with these outcomes (Figure 4). This conceptual framework has improved our initial work in five ways: it is consumer‐oriented and based on a systematic review, and it includes a harmonized typology with new (not in the initial framework) negative health outcomes and health service‐related outcomes. It is comprised of 13 main concepts vs. six initially (one was revised and seven new were added), and 42 factors and outcomes versus 30 initially (four were removed, 18 revised, and 24 new added). In contrast to existing sparse evidence, this framework integrates knowledge on health outcomes of OCHI, and establishes a chain of evidence linking ultimate outcomes of OCHI (such as health outcomes and outcomes affecting healthcare services), intermediary outcomes (such as cognitive impact and use of information), information needs and behaviors, and contextual factors.

These results contribute to information science, as few studies have examined ultimate outcomes of information such as health outcomes (Case & Given, 2016; Urquhart & Turner, 2016). Specifically, our work is a departure from previous studies that focus on a limited number of factors and outcomes, and usually only positive outcomes. In addition, our results improve substantially the initial framework in terms of conceptual comprehensiveness and clarity, which are based on a systematic review process including rigorous thematic synthesis, and an illuminating harmonization analytical step, respectively. Combining information and psychosocial theories with health research results, our framework contributes to better describe and explain OCHI outcomes and associated factors. For instance, it proposes that effects of information needs and seeking behaviors on OCHI outcomes are situational and depend on contextual factors. The framework is focused on the OCHI consumers' perspective when information is used for themselves, a loved one, or a member of their social network.

In addition to individual consumer information outcomes, this framework includes an organizational level of outcomes affecting healthcare services. This level seems increasingly relevant as most people go on the Internet to look for consumer health information (or know someone who can go online for them) and use the acquired information to make healthcare decisions (Fox & Duggan, 2013). Also, almost everyone is a primary care patient, or a relative or a family caregiver of a primary care patient, and this framework can play a large role in these individuals' healthcare and health. Although most of the OCHI outcomes mentioned in the 65 included studies were positive, we found 23 studies that reported at least one negative outcome. These negative outcomes ranged from increased anxiety to noncompliance with a healthcare management plan. At the organizational level, 12 studies suggested OCHI may lead to overuse of healthcare services, or inappropriately avoid them.

Future research on OCHI outcomes can focus on how to increase positive outcomes and prevent negative ones. For example, negative outcomes were examined from consumer, health practitioner, and librarian perspectives in a recent qualitative study (El Sherif, R, Pluye, Thoër, & Rodríguez, 2018). This study identified three types of negative outcomes: internal (for example, increased worrying), interpersonal (for example, deterioration in the patient–physician relationship), and service‐related (for example, inappropriate visits to the emergency department), then outlined potential preventive interventions. For instance, primary care professionals may pay more attention to providing trustworthy information resources and information aids to patients. Specifically, information‐related training to health professionals can be enriched with respect to the education curriculum of medical students and family medicine residents, and the continuing medical education of practicing family physicians. In addition, future longitudinal research may scrutinize the potential iterative effect of OCHI outcomes on information needs and behavior; for example, a positive outcome may improve relationships with professionals and information needs, while negative outcomes may hinder needs.

Of 65 included studies, 62 (95.4%) were conducted in OECD countries (Organization for Economic Co‐operation and Development). In OECD countries, primary care services and Internet use are aligned (Davis, Stremikis, Squires, & Schoen, 2014). Only three studies (4.6%) were conducted outside OECD countries (China, Pakistan, and Turkey; see Appendix 3 for details). Removing these studies from the synthesis did not influence the results, as no outcome was uniquely reported in these studies. This can be seen as a potential strength in terms of qualitative theoretical generalizability outside OECD countries.

### *Strengths and Limitations*

Although there is no specific method (yet) for appraising the quality of mixed studies reviews (integrating qualitative and quantitative evidence) (Bouchard, Dubuisson, Simard, & Dorval, 2011), we found that three out of four ROBIS criteria were sufficiently generic, and we applied them to critically reflect on our work (the fourth criterion being about statistical procedures for synthesizing; thus, not applicable for appraising our qualitative data synthesis). ROBIS is a recent validated method used for critically appraising systematic reviews of quantitative studies assessing the effectiveness of interventions (for example, randomized controlled trials), and etiology or diagnosis or prognosis studies, in health sciences (Whiting et al., 2016). This critical reflection suggested three strengths of our work: (a) study eligibility criteria were clear and appropriate for the review question; (b) the identification of studies was based on a librarians' exhaustive search strategy (including an appropriate range of databases/sources), and efforts were made to minimize errors in the selection of studies; and (c) the data collection and study appraisal were conducted in a rigorous manner.

Harmonizing the uncovered themes and integrating information and psychosocial theories enriched our conventional hybrid (deductive–inductive) thematic qualitative data analysis. Specifically, using harmonization for improving thematic analysis in qualitative research is original and rare: we searched the Scopus database and found no reviews and only one empirical study that used a similar method (Duracinsky et al., 2012). Because harmonization is rooted in information science and practice, it was relevant to apply this method for our review on information studies in primary care. It is applicable for any systematic mixed studies review that uses qualitative synthesis and is aimed to build a typology or developing a theory.

A limitation of our work is that it does not pertain to people who are *information poor*. The information poor (a) perceive themselves as persons who cannot be helped, (b) adopt self‐ or group‐protective behaviors, (c) are secretive and mistrust others, and (d) consider exposure to information as a risk (harm outweighing benefits; Chatman, 1996). Typically, they have low socioeconomic status, face severe mental health challenges, or live outside contemporary society, for example, for religious reasons. They have a low level of literacy, and little communication skills or social networks to help them overcome their individual literacy barrier. Few are without access (direct or mediated) to online information. Currently, only 1% of 18‐ to 29‐year‐old Americans do not access the Internet (Anderson & Perrin, 2016). As another illustration, a Quebec 2015 survey of a representative sample of 23,693 parents of preschool children showed that only 1.5% of parents do not know where to find online information about children, either directly or mediated by someone else (Lavoie & Fontaine, 2016).

Our review faced at least five other limitations. First, publication bias usually leads to an underreporting of negative outcomes, and an overreporting of positive outcomes in the literature. Although we aimed to address this issue using an exhaustive search in bibliographic and gray literature databases with the help and expertise of specialized librarians, we only synthesized current scientific knowledge on OCHI outcomes in primary care. Therefore, our results may not be transferable to health problem‐ or disease‐specific consumer information programs that are provided by hospitals and public health agencies (outside primary care). In addition, included studies were mostly centered on individual information behavior (in a social context), and our results may differ from outcomes of collective information behavior (Burnett & Jaeger, 2008; Evans & Chi, 2008); for example, one study identified the following outcomes of collective information produced by and shared between patients: finding information, feeling supported, maintaining relationships with others, affecting behavior, experiencing health services, learning to tell the story, and visualizing disease (Ziebland & Wyke, 2012).

Second, our appraisal and synthesis were limited to what was reported in the empirical studies, and we found that contextual factors, information needs, and information‐seeking behavior were rarely, or only superficially, reported in relation to information outcomes. Our work constitutes an attempt to overcome this limitation of individual studies because we pooled all study results and integrated them with information and psychosocial theories. The resulting conceptual framework and typology of OCHI outcomes are applicable in primary care settings and primary care research, and may be transferable (adapted) in neighboring domains. Third, we conducted a conventional card sorting with team members, while we could have used other methods; for example, Bussolon (2009) reports an innovative crowdsourced online card sorting with 4,100 participants (608 completed the tasks), which allowed statistical analyses. Fourth, although we did not systematically review all the theories on information behavior and health behavior, we reviewed the most common ones (including multiple reviews of these theories) and reported the theories that supported our work. Fifth, we relied on the reporting of investigators of included studies (publications) to establish an association between OCHI, outcomes, and associated factors, that is, a researchers' narrative explanation (Abbott, 1998).

### *Practical Implications*

**Figure 5:**
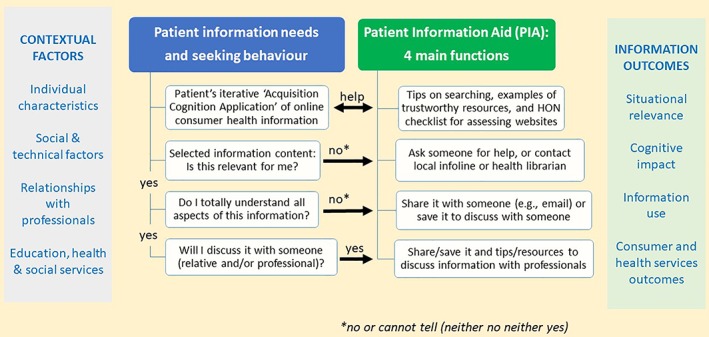
Framework‐based design of a primary care patient information aid (PIA).

The systematic review constitutes an important validation step in the development of this framework, and its use in future research will help to revise it and improve its validity. Similar to any theory, this framework may guide evaluations and interventions (Glanz et al., 2008). On the one hand, it might guide OCHI providers and professionals to document and measure the outcomes of their informational content and service, respectively, from the viewpoint of primary care information users. On the other hand, it can guide them for designing and planning interventions. For example, we used it to design a Patient Information Aid (PIA) mobile‐friendly website (Figure 5). PIA aims to (a) facilitate primary care patients' information seeking, (b) enable positive information outcomes of OCHI, and (c) reduce negative information outcomes. PIA addresses the three iterative stages of our framework: before (contextual factors), during (information needs and seeking behavior), and after information‐seeking (information outcomes). PIA will accompany consumers while they navigate these stages. A full description of the development and functions of PIA are available elsewhere (Dai et al., [Link]).

## Conclusion

The results of this review address an international priority, namely, to increase the availability of trustworthy OCHI, specifically in primary care (Industry Canada, 2009; Romanow, 2002; World Summit on the Information Society, 2005). These results make an interdisciplinary theoretical contribution by linking information science and primary care research. Our proposed conceptual framework includes a clear typology of OCHI outcomes, and advances current knowledge in these two disciplines (Figures 3 and 4). The present qualitative synthesis led to a theory for analyzing (typology) and explaining (framework) information outcomes in primary care. Future empirical research or literature reviews may improve our framework and propose testable assumptions, thereby a theory for predicting these outcomes. Such future work may scrutinize the transferability of, and eventually adapt, the proposed framework outside primary care.

Our results may be applied in evaluation, research, and practice. We identified OCHI outcomes and associated factors in the literature, which may contribute to improve information assessment methods. Future research can support or confirm these factors and outcomes using empirical research, and qualitatively explore or quantitatively measure all the proposed relationships between factors and outcomes. Better information assessment methods can assist information providers and researchers in evaluating information content retrieved from or delivered by information technology, and in managing information systems taking into account information consumers' evaluation. Specifically, the proposed cognitive, emotional, and behavioral information outcomes cast some light on what happens to individuals and their relatives when they search the Internet or use digital services and information systems. Finally, our results can help designing and planning interventions such as the PIA web application.

## Supporting information

Appendix S1: Supplementary Material.
