# Supplementary material for: Health outcomes of online consumer health information: A systematic mixed studies review with framework synthesis
Source: J Assoc Inf Sci Technol. 2019 Jan 30;70(7):643–59. doi: 10.1002/asi.24178 (PMC6686988; doi:10.1002/asi.24178)
Supplement: Supplementary file 1 — Appendix S1: Supplementary Material. [file ASI-70-643-s001.docx]

Proposed Online Appendices

- Appendix 1: Common types of qualitative syntheses for qualitative and mixed studies reviews
- Appendix 2: Search Strategy for Medline
- Appendix 3: Description of Included Studies
- Appendix 4: Harmonization of Themes: Outcomes of Online Consumer Health Information
- Appendix 5: Harmonization of Themes: Online Consumer Health Information Needs Driving Seeking Behaviour
- Appendix 6: Card Sorting Exercise: Contextual Factors

Appendix 1

Common types of qualitative syntheses for qualitative and mixed studies reviews

**An emerging type of literature reviews: Mixed studies reviews**

Literature reviews are increasingly being produced, published and cited. There is a recent increase of mixed studies reviews (MSR) (Hong, Q. & Pluye, In Press). The conceptualization of mixed studies reviews was highly influenced by the development of mixed methods research, which combines qualitative and quantitative methods in primary research using data from experimentation, observation and simulation (Creswell & Plano Clark, 2018; Pluye & Hong, 2014).

A literature review is aimed to address a specific question using research results (evidence) in a reproducible manner. The main steps of a review are: the retrieval and selection of relevant studies using clear eligibility criteria and sources of information; then, the extraction and synthesis of data (study results) from included studies using appropriate methods to answer the question. The main types of literature review can be summarized in accordance with the types of (a) review focus, (b) evidence synthesized, and (c) overall review process. First, the focus of the review may be on interventions or programs, or non-programmatic issues such as prevalence, incidence, diagnostic, prognostic, research methods, scientific theories, and so forth. MSR are usually centered on programs with a focus on participants’ viewpoints, program processes or outcomes (or foci, e.g., processes and outcomes).

Second, qualitative and quantitative evidence are based on qualitative and epidemiological/statistical methodologies and methods, respectively. This defines three types of reviews (Grant & Booth, 2009; Hong, Q., Pluye, Bujold, & Wassef, 2017; Pope, Mays, & Popay, 2007). In qualitative reviews, the question is qualitative, the included studies are based on qualitative research methods, and the synthesized evidence is qualitative (Booth et al., 2016; Gough, Thomas, & Oliver, 2012). In quantitative reviews, the question is quantitative, the included studies are based on quantitative methods, and the synthesized evidence is quantitative (Higgins & Green, 2011). In MSR, the question can be qualitative or quantitative or both, and the included studies are qualitative, quantitative and mixed methods studies, while synthesized evidence is both qualitative and quantitative (Heyvaert, Hannes, & Onghena, 2016; Hong, Q. et al., 2017).

Third, the two main types of the overall review process are the common review and the systematic review. In common reviews for research protocols and publications (e.g., a thesis), the retrieval of relevant studies to address a specific question should ideally involve a librarian, and may be performed in only the few most pertinent bibliographic databases in a specific manner (high precision) depending on time and resources available, while only one reviewer is usually involved in the selection of relevant studies, and the extraction and synthesis of data from included studies (Randolph, 2009). In systematic reviews, at least one specialized librarian establishes a comprehensive search (high recall, up to saturation), then two independent reviewers perform the selection and appraisal of included studies, and the extraction and synthesis of data (Chalmers, Hedges, & Cooper, 2002; Pluye, Hong, Bush, & Vedel, 2016). There is a variant of these two review processes, called ‘rapid review’ or ‘restricted review’ (Aronson, Heneghan, Mahtani, & Plüddemann, 2018; Tricco, Langlois, & Straus, 2017), which is a decision/policy-maker-driven question/process that can be a common or systematic review process with synthesis of qualitative or quantitative evidence, or both.

In accordance with these three types of reviews, MSR are usually focussed on a program, they include a synthesis of qualitative and quantitative evidence, and can be systematic or not. MSR face specific challenges in terms of retrieval and appraisal and synthesis. For example, a critical appraisal tool was developed to appraise the quality of different study designs: the Mixed Methods Appraisal Tool (MMAT) (Hong, Q. et al., In Press). By limiting the appraisal to core criteria, the MMAT can provide a more time efficient appraisal, and offer a practical alternative solution to the combination of different critical appraisal tools (one per type of design of included studies).

**A burgeoning area: Syntheses of qualitative and quantitative evidence**

At the end of the 1980s, researchers in social sciences were interested in synthesizing qualitative evidence in literature reviews. This led to the development of interpretative synthesis methods, which aim to generate new ways of understanding a phenomenon (Gough et al., 2012). Several interpretive (also called configurative) synthesis approaches were developed such as meta-ethnography (Noblit & Hare, 1988), meta-synthesis (Jensen & Allen, 1996; Sandelowski, Docherty, & Emden, 1997), and meta-study (Zhao, 1991). Aggregative approaches were also applied to the synthesis of qualitative evidence. For example, the centennial Content Analysis method was used to transform qualitative evidence into quantitative evidence; it consists of assigning variables/codes to qualitative results using a reliable coding grid (variables with numerical values and their definitions), and performing statistical analysis on coded data (Neuendorf, 2002).

The synthesis of quantitative evidence can address effectiveness questions such as ‘Does this program work?’ and ‘What program works for whom?’. Besides effectiveness, other questions can be ‘Why does this program work?’, ‘How does it work?’, ‘In what context and when?’, and ‘What are the factors that promote or hinder its implementation?’ These questions can be addressed by synthesizing qualitative and quantitative evidence (Petticrew, M. et al., 2013; Whittemore, Chao, Jang, Minges, & Park, 2014).

Three co-authors of the present article recently reviewed 459 systematic MSR that used multiple synthesis options (Hong, Q. et al., 2017). This showed multiple new interpretive methods for synthesizing qualitative and quantitative evidence. The most common types of qualitative syntheses of qualitative and quantitative evidence are presented Table 1. Some of the types are not mutually exclusive as the simple grouping can be combined with any other type. In addition, qualitative content analysis or thematic synthesis (higher level of interpretation of data into themes) can be combined with meta-narrative, critical interpretive, realist, or framework synthesis.

1. Meta-narrative synthesis is aimed to clarify a construct by highlighting commonalities and differences among the multiple research traditions in which it has been studied (Greenhalgh et al., 2005; Wong, Greenhalgh, Westhorp, Buckingham, & Pawson, 2013a).
2. Critical interpretive synthesis is aimed to generate theory from a large body of evidence using meta-ethnographic methods such as reciprocal translational analysis (translate concepts across included studies), refutations within and across included studies, and lines of argument (building a general interpretation for representing all studies by constant comparisons between individual studies) (Dixon-Woods et al., 2006).
3. Framework synthesis is aimed to produce a new conceptual framework based on *a priori* framework and new themes (Carroll, Booth, Leaviss, & Rick, 2013). As illustrated in the prsent paper and in Veinot, Senteio, Hanauer, and Lowery (2017), it consists of analyzing data using the *a priori* framework, creating new themes by performing thematic synthesis, and producing a new framework.
4. Realist synthesis is middle range theory-driven (a middle range theory potentially explains all similar programs, but is not a macro-theory explaining something bigger than programs, neither the micro-logical model of one individual program in one setting); it is aimed to test a candidate theory or theories, and produce a revised/improved middle range theory explaining why a program works. The latter is based on demi-regularities (patterns) identified across ‘context-mechanism-outcome’ configurations observed within included studies (Pawson, Greenhalgh, Harvey, & Walshe, 2005; Wong, Greenhalgh, Westhorp, Buckingham, & Pawson, 2013b).

Over time, qualitative, quantitative and mixed methods have been developed to address different and complementary research questions. The development of the synthesis methods applied in reviews is following a similar evolution from aggregative synthesis of quantitative evidence to interpretative synthesis of qualitative evidence, and currently the mixing of different synthesis methods and qualitative and quantitative evidence. Yet, the number of types of qualitative synthesis methodologies and methods is increasing. They are described in the table below. Researchers interested in qualitative syntheses used in MSR are invited to read Littell (2018)’s report from The Campbell Collaboration.

In conclusion, the number of MSR is rapidly increasing, and the development of synthesis methods for MSR is particularly burgeoning in health and social sciences, while qualitative and quantitative systematic reviews are commonly used in almost all disciplines (Petticrew, Mark, 2001). Finally, a wiki 'Toolkit for Mixed Studies Reviews' has been created to support a graduate course on MSR at McGill University, and may be of interest to all evaluators, students and researchers (Pluye, Hong, Granikov, & Vedel, In Press). The purpose of this wiki is to provide a practical free public step-by-step MSR-specific guidance, and suggest tips and tools for planning, conducting and reporting MSR. In addition, wiki-users are invited to provide constructive feedback and contribute to the continuing improvement of the wiki, which received more than 9,000 visits between November 2013 and September 2018 ([http://toolkit4mixedstudiesreviews.pbworks.com](http://toolkit4mixedstudiesreviews.pbworks.com/)). This wiki-toolkit is structured according to eight typical steps of systematic MSR: Question, Eligibility, Source, Identification, Selection, Appraisal, Extraction, and Synthesis (memorisable using the QESISAES acronym).

**Table 1. Common qualitative synthesis methods used in mixed studies reviews**

| **Synthesis method** | **Aim** | **Description** |
| --- | --- | --- |
| Critical interpretive synthesis | To build a theory from the synthesis of a diverse body of evidence. | Adapted the three main strategies of meta-ethnography for synthesizing qualitative and quantitative data: translating the concepts from studies into one another (reciprocal translational analysis), exploring and explaining contradictions between studies (refutational synthesis), and linking constructs and building a picture of the whole from studies (lines-of-argument synthesis). |
| Framework synthesis | To produce a new framework based on *a priori* and new themes. | Consists of analyzing data using an *a priori* framework, creating new themes by performing thematic synthesis, and producing a new framework. |
| Grouping and Clustering | To describe included studies. | Summarizes and organizes included studies into groups (categories). |
| Meta-narrative synthesis | To make sense of complex and conflicting findings by unfolding the storyline of research traditions. | Maps research traditions and consider how they have been conceptualized, theorized and empirically studied over time. |
| Narrative synthesis* | To summarize and explain the findings of included studies. | Adopts a textual approach to the process of synthesis and follows four elements: develop a theory of how the intervention works, why and for whom; develop a preliminary synthesis; explore relationships within and between studies; and assess the robustness of the synthesis. |
| Qualitative content analysis | To understand a phenomenon of interest by focussing on the content or contextual meaning of text. | Uses an analytical coding process to organize content of textual data into fewer content categories. |
| Realist synthesis | To unpack how interventions work in particular contexts through theoretical explanation (middle-range theory). | Uses theory-driven context-mechanism-outcome configurations, demi-regularities and abduction (hunches). |
| Textual description | To describe included studies. | Provides a descriptive paragraph of each study. |
| Textual narrative synthesis | To describe included studies. | Arranges studies into homogeneous groups and compares similarities and differences across studies. |
| Thematic synthesis | To identify and develop themes across included studies. | Uses line-by-line coding and, develops descriptive themes and generates analytical themes. This might lead to propose a conceptual framework. |

Legend: This table is based on a review of 459 MSR (Hong, Q. et al., 2017). Alternative typologies of qualitative synthesis methods can be found in two other publications (Booth et al., 2016; Littell, 2018).

*Narrative synthesis deserves a special note as it constitutes a meta-category including multiple types of qualitative synthesis (including textual description, grouping and content analysis for example) (Popay et al., 2006). It is usually seen as a companion synthesis to a quantitative synthesis such as: Bayesian synthesis, Configurational comparative method, Cross-design synthesis, Meta-analysis, Meta-regression, Quantitative content analysis, and Vote counting. Bayesian synthesis is used to measure the likelihood of different values for parameters of interest (incorporating prior distributions of unknown parameter values that are then updated by deriving posterior probability distributions generated through statistical analysis of the estimates). Configurational comparative method is used to test theories and assumptions by identifying configurations of causal conditions, i.e., combination of conditions that are necessary and/or sufficient for a given outcome (comparative case-oriented research method based on Boolean algebra to generate configurations between conditions and outcomes across cases). Cross-design synthesis is used to combine results from quantitative studies with complementary designs (e.g., RCT and observational studies). Meta-analysis is used to obtain a single summarized ‘effect size’. Meta-regression is used to relate the size of effect to one or more characteristics of the included studies, hereby to explore sources of heterogeneity of studies. Meta-summary is used to quantitatively aggregate qualitative findings. Quantitative content analysis is used to transform qualitative data into few variables (numerical value) for statistical analysis. Vote counting is used to calculate the frequencies of categories of results across included studies.

Appendix 2

Search Strategy in Medline

| Concept: Internet-Based Consumer Health Information | Concept: Mixed Filter To Retrieve All Types of Empirical Records | |
| --- | --- | --- |
| 1. Consumer Health Information/  2. consumer health information.mp.  3. consumer health resource*.mp.  4. consumer health.mp.  5. patient education.mp.  6. Patient Education as Topic/  7. patient education as topic.mp.  8. (patient adj1 inform*).mp.  9. decision aid*.mp.  10. decision tool*.mp.  11. decision guide*.mp.  12. decision support.mp.  13. 1 or 2 or 3 or 4 or 5 or 6 or 7 or 8 or 9 or 10 or 11 or 12  14. information utilization.mp.  15. (information adj1 us*).mp.  16. (evidence adj1 us*).mp.  17. information behavio?r*.mp.  18. information practice*.mp.  19. information seek*.mp.  20. Information Seeking Behavior/  21. information seek* behavio?r.mp.  22. shared decision making.mp.  23. (decision* adj1 support).mp.  24. search strateg*.mp.  25. 14 or 15 or 16 or 17 or 18 or 19 or 20 or 21 or 22 or 23 or 24  26. (information adj1 provi*).mp.  27. Information Services/  28. information service*.mp.  29. information centre*.mp.  30. information center*.mp.  31. resource centre*.mp.  32. resource center*.mp.  33. health information centre*.mp.  34. health information center*.mp.  35. Information Centers/  36. 26 or 27 or 28 or 29 or 30 or 31 or 32 or 33 or 34 or 35  37. 25 or 36  38. 13 and 37  39. Internet/  40. online.mp.  41. electronic.mp.  42. e-resource*.mp.  43. CD-ROM/  44. web.mp.  45. world wide web.mp.  46. Computers/  47. computer based.mp.  48. Software/  49. Computer-Assisted Instruction/  50. search engine*.mp.  51. electronic encyclop?edia*.mp.  52. online encyclop?edia*.mp.  53. 39 or 40 or 41 or 42 or 43 or 44 or 45 or 46 or 47 or 48 or 49 or 50 or 51 or 52  54. AboutKids Health.mp.  55. Canadian Cancer Society.mp.  56. Canada Safety Council.mp.  57. Caring for Kids.mp.  58. Dietitians of Canada.mp.  59. (Heart and Stroke Foundation).mp.  60. Here to help.mp.  61. PasseportSante.mp.  62. Sexualityandu.mp.  63. Women?s Health Matters.mp.  64. Cancer?gov.mp.  65. (Centers for Disease Control and Prevention).mp.  66. familydoctor*.mp.  67. familydoctor?org.mp.  68. healthfinder.mp.  69. HIV InSite.mp.  70. Kidshealth.mp.  71. Mayo Clinic.mp.  72. medem.mp.  73. MedlinePlus/  74. noah.mp.  75. new york online access to health.mp.  76. wikipedia.mp.  77. google.mp.  78. googl*.mp.  79. yahoo.mp.  80. msn.mp.  81. ask?com.mp.  82. 54 or 55 or 56 or 57 or 58 or 59 or 60 or 61 or 62 or 63 or 64 or 65 or 66 or 67 or 68 or 69 or 70 or 71 or 72 or 73 or 74 or 75 or 76 or 77 or 78 or 79 or 80 or 81  83. 53 or 82  84. 13 and 37 and 83 | 85. Case Reports/  86. Organizational Case Studies/  87. Qualitative Research/  88. qualitative study.mp.  89. action research.mp.  90. Community-Based Participatory Research/  91. participatory research.mp.  92. case stud*.mp.  93. ethno*.mp.  94. grounded theory.mp.  95. phenomeno*.mp.  96. Narration/  97. narrative*.mp.  98. biograph*.mp.  99. Autobiography/  100. documentar*.mp.  101. qualitative synthes*.mp.  102. active feedback.mp.  103. conversation*.mp.  104. discourse*.mp.  105. thematic.mp.  106. qualitative data.mp.  107. key informant*.mp.  108. Focus Groups/  109. focus group*.mp.  110. case report*.mp.  111. Interview/  112. interview*.mp.  113. Observation/  114. observer*.mp.  115. visual data.mp.  116. (audio adj record*).mp.  117. Anthropology, Cultural/  118. experience*.mp.  119. 85 or 86 or 87 or 88 or 89 or 90 or 91 or 92 or 93 or 94 or 95 or 96 or 97 or 98 or 99 or 100  or 101 or 102 or 103 or 104 or 105 or 106 or 107 or 108 or 109 or 110 or 111 or 112 or 113 or  114 or 115 or 116 or 117 or 118  120. exp clinical trial/  121. randomized controlled trial/  122. controlled clinical trial/  123. exp Research Design/  124. random allocation/  125. double-blind method/  126. Single-Blind Method/  127. Placebos/  128. Cross-Over Studies/  129. 120 or 121 or 122 or 123 or 124 or 125 or 126 or 127 or 128  130. clinical trial*.mp.  131. (clinic* adj25 trial*).mp.  132. random*.mp.  133. control*.mp.  134. controlled trial*.mp.  135. (latin adj square).mp.  136. placebo*.mp.  137. 130 or 131 or 132 or 133 or 134 or 135 or 136  138. Comparative Study/  139. Validation Studies/  140. evaluation studies/  141. Follow-Up Studies/  142. Prospective Studies/  143. Cross-Over Studies/  144. prospective*.mp.  145. volunteer*.mp.  146. 138 or 139 or 140 or 141 or 142 or 143 or 144 or 145  147. singl*.mp.  148. doubl*.mp.  149. trebl*.mp.  150. tripl*.mp.  151. 147 or 148 or 149 or 150  152. mask*.mp.  153. blind*.mp.  154. 152 or 153  155. 151 and 154  156. (animal not human).mp.  157. 129 or 137 or 146 or 155  158. 157 not 156  159. Cohort Studies/  160. Case-Control Studies/  161. Cross-Sectional Studies/  162. Health Surveys/  163. Health Care Surveys/  164. Risk/  165. Incidence/  166. Prevalence/  167. Mortality/ | 168. "case series".mp.  169. "time series".mp.  170. "before and after".mp.  171. prognos*.mp.  172. predict*.mp.  173. course*.mp.  174. 159 or 160 or 161 or 162 or 163 or 164 or 165 or 166 or 167 or 168 or 169 or 170 or 171 or  172 or 173  175. mixed method*.mp.  176. mixed-method*.mp.  177. (mixed adj5 method*).mp.  178. multimethod*.mp.  179. multiple method*.mp.  180. (multiple adj5 method*).mp.  181. 175 or 176 or 177 or 178 or 179 or 180  182. qualitative.mp.  183. Qualitative Research/  184. quantitative.mp.  185. (quantitative adj5 research).mp.  186. 182 or 183  187. 184 or 185  188. 186 and 187  189. 181 or 188  190. 119 or 158 or 174 or 189  191. 84 and 190  192. limit 191 to yr="1990 -Current" |

Appendix 3

Description of Included Studies

| First Author (Year) Study ID | Design, Setting, Participants and Data Collection | Contextual Factors | Types of Information Use | Types of Health- And Health Care- Related Outcomes | MMAT Score |
| --- | --- | --- | --- | --- | --- |
| (Ahmad, Hudak, Bercovitz, Hollenberg, & Levinson, 2006) | Qualitative descriptive study: Six focus groups were conducted with 48 family physicians practising in Toronto (Canada). | eHealth literacy  “Physicians attributed patient confusion to their limited ability to evaluate, personalize, and interpret abundant Internet health information.” | Discussion with health professional  “Physicians in our study generally perceived Internet based health information as problematic when introduced by patients during medical consultations.” | Outcome for relationship with health professional  “Internet health information resulted in patient distress, which was perceived by physicians as patient “anxiety,” “worry,” “nervousness,” panic,” or the patients feeling “overwhelmed” or “sicker”. (…) In having their expertise challenged, some physicians felt they were at risk of “losing face” and/or being “put on the spot”. (…) They discussed having limited time to deal with Internet-derived “volumes of pages” or “scrolls” of questions that patients “bring to their visits.”  Health professionals’ outcomes  Some physicians discussed strategies of “firing” the patient, referring patients to specialists, or charging for extra time. These strategies have the potential to undermine the physician-patient relationship. | 75% |
| (Amirault et al., 2005) | Prevalence survey (quantitative descriptive): Study participants were Nova Scottia Health Net website users who completed and submitted an online survey questionnaire between June 2002 and June 2003 (N = 114). The majority of respondents were white, middle-aged, English-speaking females, residing in rural Nova Scotia (Canada). | Level of education  “99% of the sample indicated that they had a high school diploma as a minimum level of education; 20% reported having a community college education, 34% an undergraduate university education, and 22% a graduate degree.”  eHealth literacy  “83% of users reported that it was easy or very easy to find the information they wanted (64% and 19%, respectively).” | Discussion with health professionals  “Fifteen of the respondents (32.6%) indicated that they would be sharing the information with their health care provider.”  Use for change of health management  “11 people (22.91%) indicated that they planned to use the information to help change their lifestyles. Twenty participants (37.03%) indicated that this information would be used to provide or influence care.”  Use for providing social support  “63 (80.76%) people indicated they were going to share the information with others.” | Outcome for problem management  “24% stated they used the information to decide to go to the doctor [family physician], and another 8% indicated that the information had helped them to decide not to go to the doctor.” | 50% |
| (Anderson, 2004) | Prevalence survey (quantitative descriptive): Computer-assisted telephone interviews of a national random sample of 3,000 phone numbers. Only 186 adults were interviewed (USA). | Professionals’ attitude  “Significant relationships were found between an individual’s belief that their doctor would be upset with them for having sought information on the Internet and race.”  Confidence in OCHI  “Belief in the Internet’s reliability positively affects whether an individual has discussed information with his or her doctor and the belief that such an action would upset their physician.” | Discussion with health professional  “Only a little more than a third of respondents (36%) who used the Internet for health-related purposes had discussed with their provider information about illness or treatments they had obtained online.” | Outcome for relationship with health professional  “Almost 1 out of 10 respondents felt that their physician would be upset if they used the Internet and the Web to investigate their health conditions, 16% were uncertain how their physician would respond.” | 50% |
| (Ayers & Kronenfeld, 2007) | Non-randomized cross-sectional survey (quantitative non-randomized): The data for this study were derived from Lo’s study on the ‘Impact of the Internet and Advertising on Patients and Physicians, Report 2000–2001’ (University of California, 2004). The sample size was 3009 respondents in the ﬁrst analysis, and 1179 in the second analysis (USA). | Chronic disease  “The ﬁndings suggest that it is not merely the presence of a particular chronic illness, but rather the total number of chronic conditions that determine Internet use for health information.”  Age and Level of education  “As education and income increase, the frequency of using the Internet to retrieve health information increases.” | Use for change of health management  “Also, the more frequently a person uses the Internet as a source of health information, the more likely they are to change their health behavior.” | Outcome for involvement  Lastly, of those who used the Internet to retrieve health information, 53.6% did not change their health behavior, while 46.4% did change their health behavior. | 75% |
| (Baker, Wagner, Singer, & Bundorf, 2003) | Prevalence survey (quantitative descriptive): A survey was conducted in December 2001 and January 2002 among a sample drawn from a research panel of more than 60,000 American households. Responses were analyzed from 4764 individuals aged 21 years or older who were self-reported Internet users (USA). | Level of education  "In particular, we find that individuals with less education are less likely to use the Internet for health care.”  Chronic disease  “Among those without any of the 5 chronic conditions, 67% said that use of the Internet improved their understanding of health care issues.” | Use for decision-making  “67% said that use of the Internet improved their understanding of health care issues, fewer respondents said that the Internet affected more substantive decisions or activities such as improving their ability to manage their health care needs on their own or influencing their choice of health care professional.” | Organizational outcomes  “About one third of those using the Internet for health reported that using the Internet affected a decision about health or their health care, but very few reported impacts on measurable health care utilization; 94% said that Internet use had no effect on the number of physician visits they had, and 93% said it had no effect on the number of telephone contacts.” | 100% |
| (Bansil, Keenan, Zlot, & Gilliland, 2006) | Prevalence survey (quantitative descriptive): The study population was selected from respondents to the 2002 and 2003 HealthStyles surveys, which included a series of questions about participants’ use of the Internet for accessing health-related information and their health status (N = 8432) (USA). | Age, gender, level of education  Younger people, women, people with at least a college degree, and people who earned $60,000 or more annually were more likely to seek health information online.  Health status  Internet users with one or more reported chronic disease, or with depression and high cholesterol were more likely to seek health-related information on the Web compared with those without these conditions. | Discussion with health professionals  “Among respondents who reported accessing health information on the Web, 53% reported sharing the information with their physicians sometimes; 11%, usually; 3%, always; and 32%, never.” | No outcomes reported. | 75% |
| (Beck et al., 2014) | Prevalence survey (quantitative descriptive): Authors reported the French Health Barometer 2010 survey of a nationally representative sample of 1052 young adult Web users aged 15-30 years (France). | Confidence in OCHI  Approximately 80% of online health seekers trusted the information they found on the Internet, even if 61.4% of them qualified the information only as “somewhat” reliable, without significant differences according to age and gender. | Use for change of health management  “Finally, although 26.6% looked for online health information without having had any kind of medical consultation, 33.1% reported they modified the way they take care of their health based on the information they found on the Internet.” | Outcome for involvement  “Moreover, a total of 33.1% of the 15-30 years group of health seekers stated they changed their way of taking care of their health” as a result of OCHI.  Organizational outcomes  For 11.4% of young online health seekers, the information found on the Internet in the past 12 months led them to see a doctor [family physician] more often or less often (6.5%) than usual: the 20-25 years group tended to see their doctors less frequently (9.9%) than the 15-19 years (4.1%) and the 26-30 years (4.7%) groups. | 100% |
| (Berg, 2011) | Sequential mixed methods design: Data were gathered in a larger multidisciplinary research project conducted in East York, Ontario. An initial statistical analysis of 350 survey responses described Internet usage. A qualitative analysis was conducted on 86 follow-up interviews of people recounting their personal health management processes (Canada). | eHealth literacy  “I would say that by and large I know how to pick sites that are good versus sites that aren’t.” | Use for discussion with a health professional  “Doing research before a doctor’s visit to be able to discuss information found, get a second opinion, form questions for the visit.” | Outcome for relationship with health professional  “She uses Internet research as expertise to question her doctor, feeling prepared in advance offers Hilary (alias) more control on the outcome of the doctor patient interaction and possibly the relationship.”  Other consumer health outcome “We found a connection between having current health information and pursuing new knowledge with being healthy and achieving positive health outcomes.” | 75% |
| (Bianco, Zucco, Nobile, Pileggi, & Pavia, 2013) | Prevalence survey (quantitative descriptive): The sample consisted of 1544 adults aged 18 years or older selected among parents of students in 10 public school in the geographic area of Catanzaro in southern Italy. A total of 1039 parents completed the questionnaire between April and June 2012 (Italy). | Satisfaction with professionals’ behaviour  “The behavioral discrepancy between searching for information on the Internet and not using this information with health professionals might be because of user conflict derived from not trusting health professionals whose attitude and behavior are incompatible with the information from the Internet.” | Use for discussion with a health professional  “Among participants who used the Internet to search for health-related information, only 25.4% discussed this with their GP [family physician].” | Outcome for relationship with health professional  A total of 78.5% (581/740) of the eligible respondents believed that Internet use had not changed their relationship with their GP in any way; 13.4% (99/740) believed it had a positive effect, and 8.1% (60/740) believed it had a negative effect.  Organizational outcomes  After using the Internet, 12.7% (94/740) of the sample had reduced their frequency of GP visits.  Outcome for involvement “In particular, 57.8% (296/512) reported they had become more interested in health issues, and 36.7% (188/512) were less confused about health problems.” | 100% |
| (Bujnowska-Fedak, Staniszewski, & Steciwko, 2007) | Prevalence survey (quantitative descriptive): A telephone survey was performed with 1027 people between 15 and 80 years old who were selected from a random sample of the population (Poland). | No factors reported. | Use for decision-making  The most likely use of the Internet identified in this study were: willingness to change diet or other lifestyle habits (53% of positive answers), and suggestions or queries on diagnosis or treatment to their physician (49% of positive answers). | Outcome for health improvement or worsening  39% of respondents reported feelings of reassurance or relief after obtaining information on health or illness from the Internet. | 50% |
| (Burton-Jeangros & Hammer, 2013) | Qualitative descriptive study: This study was conducted in French speaking Switzerland between 2008 and 2009. Semi-structured interviews were conducted with 50 pregnant women aged 24-41 years (Switzerland). | No factors reported. | Use for confirmation  “Internet can also be seen as a means to verify or control the information given by the doctor. The consultation specialized medical sites or articles from medical journals and is akin to the quest for a second opinion.” | Outcome for health improvement or worsening  Anxiety over the risks mentioned in the information.  Outcome for involvement  Empowerment and autonomy. | 25% |
| (Bylund et al., 2007) | Prevalence survey (quantitative descriptive): Participants were recruited from Internet health message boards, and completed an online survey (N = 770), including questions focusing on a recent interaction with a provider about Internet-based health information (USA). | Integration of OCHI in encounters with professionals  “Our data indicates that providers’ responses to patients are associated with the strategies patients use for introducing Internet information, the provider was more likely to respond [to these strategies] with active agreement.” | Discussion with health professional  “Patients reported using a variety of strategies to introduce Online health information during visits with a provider.” | Outcome for satisfaction with care, Outcome for relationship with health professional, Health professionals’ outcomes, and Outcome for health improvement or worsening  “Patients expressed the lowest level of satisfaction (average rating 2.43), validation (2.25), and reduced concern (0.41) when the provider disagreed with, and offered no validation of, the patient’s efforts in researching” OCHI. | 50% |
| (Caiata-Zufferey, Abraham, Sommerhalder, & Schulz, 2010) | Qualitative descriptive study: 27 patients, who searched for health information online before or after a medical consultation, were recruited in the waiting room of 14 medical practices to participate in semi-structured interviews between 2005 and 2007 (Switzerland). | OCHI characteristics  “The Internet was perceived by all the interviewed patients as an accessible, convenient, and exhaustive tool. Participants appreciated the fact that it was easy to use, cheap, available at any time, rapid, and anonymous.”  eHealth literacy  “They had access to it and time to use it; they knew its potential; they knew how to use it or whom to ask for help in case they were unfamiliar with it; they were able to find the information needed and to understand it.”  Social network  “The two persons who were not familiar with the Internet could count on someone in their immediate circle to help them or even make the search for them.” | Discussion with health professional  OCHI was used “to reduce uncertainty, to challenge the consultation, to prepare for a physician encounter and to discuss the information with a physician.” | Outcome for involvement  “Health information seeking (…) was considered to provide patients with the tools to play an active role during the consultation.”  Outcome for health worsening “Gaps or discrepancies in patients’ knowledge systems created or enhanced a sense of uncertainty of their situation, left them disoriented and powerless, unable to decide how to act and to protect themselves in the future.” | 75% |
| (Campbell, 2009) | Pre-post quasi-experimental survey (quantitative non-randomized): 36 self-selected participants completed the *Elderly and the Internet* program at two low-income minority residential buildings in Pittsburgh; and their responses to pre- and post-training program evaluation questionnaires were analyzed (USA). | No factors reported. | Use for decision-making  Out of 19 respondents who used the information, 8 (42%) reported that the found information enabled them to manage their health without visiting a health professional.  Use for change of health management  Fifteen out of 19 (79%) reported being "challenged" by the information to change their lifestyle (e.g., diet or exercise). | Outcome for involvement  Nineteen out of 36 (53%) used the Internet for health information after the training.  Organizational outcomes  Five out of 19 (26%) used the information to change a health care provider or facility.  Outcome for problem management  Twelve out of 19 participants (63%) reported having changed their treatment based on the found information. | 75% |
| (Chung, 2013) | Prevalence survey (quantitative descriptive): This study used data from the 2007 Health Information National Trends Survey (HINTS), developed by the National Cancer Institute (N = 5,078 Internet users) (USA). | Age and Gender  "Controlling for other factors, the middle aged (30–49 years old), compared with those who were 65 years or older, were more likely to discuss online search results with HCPs [*health care providers*]. Women, compared with men, were less likely to mention their Internet search to HCPs.” | Discussion with health professional  Among those who had gone online for health information and had seen HCPs during the previous year, 34.9% reported having discussed the searched information with HCPs. | Health professionals’ outcomes  “Reactions of HCPs to online information were perceived as particularly negative by certain groups of patients, such as those who experienced poor health and those who had more concerns about the quality of their searched information.” | 50% |
| (Coberly et al., 2010) | Case-control study (quantitative non-randomized): 426 adult patients (aged 18 years or older) with Internet access were invited to participate in an intervention study evaluating an email information prescription for selected medical conditions with links to MedlinePlus (USA). | No factors reported. | Discussion with health professionals  Out of 101 respondents in the intervention group, 52 (61%) discussed the health information from the Internet with their health care provider. | Outcome for involvement and Organizational outcomes  According to anecdotal data from 5 out of 10 participating physicians, the information prescription encouraged compliance and improved understanding of concept, procedures, and medications. | 50% |
| (Cohall et al., 2011) | Prevalence survey (quantitative descriptive): Authors reported a cross-sectional random digit-dial landline phone survey (N = 646) of adults over 18 years old in Harlem (USA). | Health status  “Indeed, current Internet users in Harlem were more likely to self-report their health as being excellent, very good, or good compared with non-current users in Harlem.” | Discussion with health professionals  Almost 53% discussed information with their health care providers. | Outcome for problem management and organizational outcome  “Of those who reported searching online for health information, 74 % sought information on medical problems and thought that information found on the Internet affected the way they eat (47%) or exercise (44%). For example, one-third reported that the information found on the Internet had improved their ability to manage their health care needs without visiting a health care provider (36%) and had led them to seek care from different health care providers than they otherwise would have (33%).” | 75% |
| (Couper et al., 2010) | Prevalence survey (quantitative descriptive): This cross-sectional survey was conducted between November 2006 and May 2007 with 2575 English- speaking US adults (aged 40 years or older), who had either undergone 1 of 9 medical procedures or tests or talked with a health care provider about doing so during the previous 2 years (USA). | Age  Internet use was higher at younger ages, rising from 14% among those aged 70 years and older to 38% for those aged 40 to 49 years. | Use for decision-making  “Use of the Internet for information related to specific decisions among adults 40 years and older was generally low (28%), but varied across decisions, from 17% for breast cancer screening to 48% for hip/knee replacement.” | No outcomes reported. | 75% |
| (Diaz et al., 2002) | Prevalence survey (quantitative descriptive): 512 patients from an internal medicine private practice were mailed a confidential survey (USA). | OCHI characteristics  Internet was perceived as more convenient and less expensive than a health care provider to get health advice from. | Discussion with health professionals  59% did not discuss the information with their health care providers.  Use for decision-making and Use for confirmation  11% used it instead of seeing or speaking with their health care provider. 69% used it for a second opinion. 83% used it for alternative or complimentary medicine. | No outcomes reported. | 50% |
| (Dolan, Iredale, Williams, & Ameen, 2004) | Prevalence survey (quantitative descriptive): The sample consisted of adult patients (N = 851) from two general practice populations at different levels of the socioeconomic spectrum in South Wales who were surveyed by questionnaire about the health information they use, including information from the Internet (UK). | Age  Patients aged 24–54 were more than twice as likely (25%) to use the Internet for health information than those aged 55 and above.  Gender In general, female patients used information sources more frequently than males during the previous 6 months. | Discussion with health professionals  About a quarter (24%) of health Internet users had discussed information during a consultation with a health professional. This information was accessed from the Internet by patients before the consultation. | Outcome for involvement and Organizational outcomes Only a small proportion (20%) had introduced information that was new to the health care professional, or felt that the consultation time increased as a result of discussing Internet information. Of those patients, most (76%) felt more prepared for a consultation by accessing the Internet. The majority also felt able to participate more in decision-making (70%) when they discussed Internet information. | 100% |
| (Ettel, Nathanson, Ettel, Wilson, & Meola, 2012) | Prevalence survey (quantitative descriptive): 497 high school students from grades 9 through 12 at a private Catholic high school participated in an anonymous cross-sectional survey,and were asked about their use of electronic tools to obtain health information, topics of interest, sources used to obtain information, and modiﬁcations in their behavior based on that information (USA). | No factors reported. | Use for change of health management  "22% of students reported modifying their behavior on the basis of their ﬁndings in online searches.”  Discussion with a health professional  “Students conducted e-mail conversations with teachers about health-related topics, but few students used this tool to communicate with their physicians." | No outcomes reported. | 75% |
| (Fox, Rainie, & Horrigan, 2006) | Prevalence survey (quantitative descriptive): Authors reported a survey on Internet use of a random digit sample of telephone numbers (N = 12,751) selected from telephone exchanges in the continental USA. | OCHI characteristics  It is important for health seekers that the information is convenient for them, that they can get more health information online than they can get from other sources, and that they can get this information anonymously. 16% of health seekers said they had used the Web to get information about a sensitive health topic that is difficult to talk about. | Use for change of health management  “47% of those who sought health information for themselves during their last online search say the material affected their decisions about treatments and care. Half of these health seekers say the information influenced the way they eat and exercise. 70% said the Web information influenced their decision about how to treat an illness or condition.”  Discussion with a health professional  “50% said the Web information led them to ask a doctor new questions or get a second opinion from another doctor.”  Use for decision-making “28% said the Web information affected their decision about whether or not to visit a doctor.” | Outcome for problem management  “48% of these health seekers say the advice they found on the Web has improved the way they take care of themselves”. | 100% |
| (Gauld & Williams, 2009) | Prevalence survey (quantitative descriptive): 406 respondents had used the Internet for health information and completed a questionnaire about emailing physicians, Internet searching, taking Internet information to the physician and perceptions of Internet information reliability (Australia and New Zealand). | eHealth literacy  Those who had taken Internet information to their physician were more likely to have been frequent Internet health information searchers, to have found information on the Internet that their physician had not previously provided, and to have frequently checked the credentials of Internet information providers. | Discussion with a health professional  Frequent Internet searchers were more likely to take OCHI to their family physician. | No outcomes reported | 75% |
| (Harbour & Chowdhury, 2007) | Cross-sectional analytic study (quantitative non-randomized): This study was based on a survey of two samples of population in Glasgow – a group of 64 OCHI users from the general public and a group of 24 post graduate students from a university in Glasgow, Scotland (UK). | Gender  Men were more likely to use the Internet (21 %) for health information.  Age Interestingly the student population reported more difﬁculties in ﬁnding online health information. This could be the result of more speciﬁc queries by students or less willingness to accept information without ascertaining quality and usability. | Use for decision-making  About two-thirds (61%) of the general public had taken actions as a result of information found on the Internet: the most common ones were visiting a family physician or changing diet. | Outcome for problem management and Outcome for involvement  The most frequently cited beneﬁts of OCHI were noted to be increasing understanding, prompting action, providing reassurance, and encouraging self-help. | 50% |
| (Hardey, 2001) | Two-phase mixed methods sequential design: Phase-1 was a qualitative study of 10 households that used home computers to find health information on the Internet. Phase-2 examined 132 home pages that contained accounts of ill health, and 74 authors’ home pages responses to an emailed questionnaire (UK). | eHealth literacy  “I found sites from all over, you know, medical stuff and some really loony ideas someone has put on their web page.”  “Most advice showed that there was not a big risk of autism and that the benefits were far more. I looked at papers from medical journals and other reports.” | Discussion with a health professional  “My GP [family physician] is very busy and does not have time to answer questions fully. Actually, it is much easier to think about what you want to ask when you look things up on the Net. I don’t get that nagging feeling that I’m needlessly taking up his time.”  Use for decision-making Make a decision about giving their child MMR vaccine after reading information on links to autism. | Outcome for relationship with health professional  This suggests the potential transformation of the physician-patient relationship (somewhat ending the medical monopoly over medical information). | 50% |
| (Hart, Henwood, & Wyatt, 2004) | Qualitative descriptive study: This study was based on interviews with, and observations of patient–practitioner interactions, of a purposive sample of 47 patients (32 women and 15 men) in the UK. | eHealth literacy  Amongst participants, many had very few information literacy skills and others lacked general computer literacy skills and/or Web-searching skills. Most of those who wanted to access information from the Web relied on intermediaries. | Discussion with a health professional  “… and patients coming in armed with printouts [of OCHI they had found] were mentioned by a further two [physicians].” | Outcome for relationship with health professional and Health professionals’ outcomes  "Patients reported that some health practitioners sought to assert their authority by dismissing the patient's acquired knowledge. For example, one woman said some health practitioners had made it clear that they thought she should not look things up for herself. She felt that the view was “you're here with me now and I'm telling you this”.”  Outcome for health improvement or worsening  “The Internet's role in feeding the anxieties of patients with hypochondria was raised by three practitioners, and problems with “all sorts of odd websites” and patients coming in armed with printouts were mentioned by a further two." | 25% |
| (Hong, T., 2008) | Prevalence analytic survey (quantitative non-randomized): Data from the National Cancer Institute’s Health Information National Trends Survey 2005 were analyzed. Two sets of logistic regression analyses were conducted, one for a subsample of Internet users (n = 3,244) and one for a subsample of Internet users who are first generation immigrants (n = 563) in the USA. | Ethnicity and socioeconomic status  Among all Internet users, Whites had higher levels of patient–provider communication about Internet health information than Blacks and Asians. Similarly, among Internet users who are immigrants, Whites had higher levels of patient–provider communication about Internet health information than Blacks and Asians. | Discussion with a health professional  Approximately 48% of Internet users and 51% of Internet users who are first-generation immigrants reported having patient–provider communication about OCHI. | No outcomes reported. | 100% |
| (Houston & Allison, 2002) | Cross-sectional analytic survey (non-randomized study): A national random-digit telephone survey was conducted by the Pew Internet & American Life Project with Internet users (N = 521) who go online for health care information (USA). | Health status  After adjustment for age, education and income, those in fair/poor health (N=59) were relative newcomers to the Internet, but tended to use the Internet more frequently, were more likely to use online chats, were less likely to search for someone other than themselves and were more likely to talk about the new information with their physician, compared with those in excellent/good health. | Discussion with a health professional  Those with fair/poor health were more likely to search for specific information on their physician and medications and were more likely to speak to their health care provider about OCHI. | No outcomes reported. | 75% |
| (Iverson, Howard, & Penney, 2008) | Prevalence survey (quantitative descriptive): a standardized eight-question survey regarding Internet use and healthcare was given to patients at three osteopathic primary care medical clinics (N = 154) in the UK. | Age  “More than half the Internet users responding to the present survey reported that the online information they found changed the way they thought about their health, with these changes most prevalent in the 31-to-45-years age group and least prevalent in those aged 61 years and older.”  Professionals’ attitudes  “Of the nine Internet users who changed their behavior but did not tell their physicians, only one user cited a feeling that the physician would disapprove of the information.” | Discussion with a health professional  “By and large, these patients informed their physicians of these changes [changes in the way they thought about their health] (30 [73%]), especially as they believed physicians were willing to discuss the health information they obtained online (75 [84%]).”  Use for change of health management  “Changed dietary habits (22 [54%]). Other notable behavioral changes reported were increased physician visits (15 [37%]) and increased use of herbal products or dietary supplements (11 [27%]).” | Outcome for involvement  “Many individuals reported increased interest in their health as a result of their independent Web-based research. The behavioral changes most commonly reported among Internet users were those that signify more active engagement during physician office visits, particularly asking more questions (27; 66%). In addition, these patients reported greater adherence to physician advice (22; 54%).”  Outcome for relationship with health professional and Health professionals’ outcomes “One major concern among physicians is the possible influence of the Internet on the patient-physician relationship, specifically insofar as patients may come to view physicians as less of an authority on medical issues. However, our survey results lead us to reject this hypothesis because—among Internet users who changed their behavior based on information they obtained online—the majority of respondents believed that their physicians would be open to discussing the health-related information they located online (84%) and shared the results of their research with their physicians (73%).” | 75% |
| (Kavathe, 2009) | Prevalence survey (quantitative descriptive): 521 people completed a structured questionnaire distributed to 45,000 Internet users in 21 counties in Northeast Ohio, USA. | eHealth literacy  “Results also showed that health seekers are more likely to choose sources of online health information that can generally be considered reliable.”  Health status “The study showed that people are more likely to access online health information if they suffer from chronic disease or disability, if their loved ones suffer from chronic disease or disability and if they suffer from nagging health.”  Confidence in OCHI “The scores indicate a moderate to moderately high levels of perceived credibility and reliability health information websites.” | Discussion with a health professional  “Health seekers reported their wish to discuss information with their healthcare providers, share it with their family and friends, and take the time to understand the information they were accessing and consider what the implications could be.” | Outcome for health improvement or worsening  “Health information from online sources is a significant component of people’s self-health plan, and results show a positive relationship between health-related behavior modifications and online health information. On the other hand, respondents scored lower on Anxiety Related to Credibility and Reliability, signifying a negative outcome expectancy, which shows that they are aware of the pitfalls of online health information.”  Outcome for involvement “These scores suggest that health seekers have a high level of both cognitive and behavioral involvement. These include (…) behavioral involvement with decision making about medical tests and treatment options. High levels of involvement are indicative of higher levels of interest in and attention to health information, which in turn signify that health seekers may want more of a say and more control of their personal health and wellness.” | 75% |
| (Kavlak, Atan, Güleç, Öztürk, & Atay, 2012)* | Prevalence survey (quantitative descriptive): 185 pregnant women (in at least the 28th week of pregnancy) from two hospitals completed a questionnaire (Turkey). | Health status  “About 45.4% of the women stated that they used the Internet throughout pregnancy to obtain information.” | Discussion with a health professional  “Fifty-one percent of the pregnant women stated that they shared the information which they had obtained on the Internet with health professionals.” | No outcomes reported. | 100% |
| (Khechine, Pascot, & Premont, 2008) | Prevalence survey (quantitative descriptive): English-speaking patients suffering from long-term diseases and using the Internet for health-related concerns completed an 18-item online questionnaire (N = 121) in Canada. | Chronic disease  Most patients from the sample use the Internet at the stages of treatment identiﬁcation (94.2%) and treatment application or follow-up (86%).  eHealth literacy  “It is important to remember that whatever the language used, patients are often more conﬁdent about the information that they get from scientiﬁc websites.” | Use in decision-making  “Concerning the categories of medical websites that patients visit the most at each stage of the decisional process, [results] show that information retrieved from online discussion groups and commercial websites is the least used by patients at all stages of the decisional process.” | No outcomes reported. | 75% |
| (Kim & Kim, 2009) | Prevalence survey (quantitative descriptive): A 25-item online questionnaire was emailed to physicians in order to determine their perception about the effects of Internet-based health information on physician–patient relationship (N = 493) (Korea). | Satisfaction with professionals’ behaviour  One hundred and seventy-nine (36.3%) of the respondents reported that they had time to discuss the Internet health information with their patients; however, 261 (52.9%) reported that there was not enough time. | Discussion with a health professional  “446 (90.5%) of the respondents replied that in the last year, their patients made inquiries about OCHI, reﬂecting that majority of the physicians have had this type of experience.” | Outcome for relationship with health professional  “Around 38% of respondents felt that Internet health information damage good physician–patient relationship. About 42.6% of the respondents felt neutral on the effect of Internet health information on the physician–patient relationship during discussion with the patient about the information. Among them, 25.6% felt the effect was negative and a less 16.6% felt that the effect was positive.”  Organizational outcomes  “Also, when asked about such discussion’s effect on the effectiveness of visits, 40.2% felt that it serves as a hindrance and only 13.6% felt that it enhances the effectiveness; and 54.8% answered that Internet health information discourages patients to follow treatment instructions or advice from their physicians. When asked if Internet health information contributes to rising of healthcare cost, 56.2% agreed. About 60.9% agreed that Internet health information causes patients to take up more of the physician’s time and 44.1% replied that Internet health information promotes unnecessary visits to physician.” | 50% |
| (Kivits, 2006) | Qualitative descriptive study: Interviews were conducted by email with 31 health information seekers (UK). | eHealth literacy  “Interviewees choose online sources either by judging the value of information by the reliability and reputation of the online source.”  Professionals’ attitudes  “Participants generally share a negative perception of how their doctors [family physicians] would react to the fact that they use the Internet to ﬁnd health information. They prefer to be silent, asking questions and discussing information, but not revealing that they use the Internet.” | Discussion with a health professional  “Either by discussing information they have accessed online or setting questions in advance, interviewees mention being able to understand better and participate in consultation sessions with their doctors by preparing for the encounter, as an effective outcome of their online information seeking. On the one hand, this is veriﬁed by two interviewees who admit sometimes contesting doctors’ diagnoses or treatments after acquiring information gathered on the Internet.” | Outcome for relationship with health professional and Health professionals’ outcomes  The result for these information seekers is sometimes an improved interaction with their physicians. Mary [alias] also uses expert and detailed information gathered on the Internet to obtain speciﬁc treatments, which has resulted in a good relationship with her gynaecologist, but a deteriorated one with her family physician. | 50% |
| (Laflamme, 2003) | Prevalence survey (quantitative descriptive): The East Baltimore Internet Health Survey (EBIHS) is a community based participatory research with 520 participants in the East Baltimore Empowerment Zone (EB-EZ), a majority low- income inner-city community. The street-intercept survey method and simple random cluster sampling were used. A survey team of trained community residents conducted the Palm®-based survey (USA). | Ethnicity and socioeconomic Status  “Several significant differences in the types of health information sought by low-income Internet users were identified.”  Level of education  “Internet users were more likely to report a higher level of education and self-efficacy, and lower values of chance health locus of control and perceived health competence.”  Gender  “Online health information seekers were more likely to be female and to have greater levels of religiosity and perceived health competence.” | Discussion with a health professional, Use for providing social support, Use for decision-making  “Fifty-six percent [of OCHI seekers] later talked with a doctor [family physician] or a nurse about the information they found online; 59% reported the information they found affected their decisions about health treatments or the way they help care for someone else. Almost half (48%) said that online health information affected their decision about whether to see a doctor. Seventy-two percent of OHS reported that the information they found online led them to ask a doctor new questions, or to get a second opinion from another doctor.” | Outcome for problem management  Sixty-seven percent of respondents said that getting OCHI improved the way they took care of their health "a lot" or "some"; 22% "only a little”; and 11% "not at all". | 75% |
| (Lagan, Sinclair, & Kernohan, 2011) | Qualitative descriptive study: 13 asynchronous online focus group were conducted across ﬁve countries over a 3-month period (N = 92). Participants were drawn from a population of 193 women who had participated in a websurvey, used the Internet for seeking information on pregnancy, and expressed a willingness to engage in a focus group (UK). | eHealth literacy  “In general, they reported trusting information from reputable sources such as ‘‘government’’ or ‘‘hospital’’ websites. Some considered whether or not the organization presenting the online information had anything to gain ﬁnancially, and if it had, they were less likely to trust that speciﬁc source. Communication, and was particularly important when women wanted conﬁdential advice, to be free to ask questions, and receive support without fear of identiﬁcation and judgment.” | Discussion with a health professional  “Most women reported discussing the information with a health professional. The main uses of the information were to validate information, aid empowerment, and assist decision-making.”  Use for providing social support “They used online discussion forums to gain support from other pregnant women or mothers, which in some instances was in the context of social isolation.” | Outcome for health improvement  “By connecting with other women, participants were able to get a more realistic picture of what was ‘normal’ and felt reassured when they were able to conﬁrm that the symptoms they were experiencing were typical of pregnancy. Many women talked about the information they acquired, that it made them feel ‘empowered’, ‘in control’, and ‘informed’, and gave them strength and conﬁdence to speak to health professionals as ‘‘an equal’’. The women in this study mainly viewed the Internet as a positive resource, it did come under some criticism. It was blamed for ‘scare mongering’.” | 75% |
| (Lev, 2009) | Qualitative descriptive study: Interviews were conducted with 50 pregnant women (one fetus and normal pregnancy) who utilized the Internet during their pregnancy (Israel). | Professionals’ attitude  “I think that, in general, physicians do not like the fact that pregnant women search for information on the Internet because the Internet is not a reliable source and the physician has studied medicine.”  eHealth literacy  “Pregnant women who utilize the Internet during their pregnancy recognize that the variable reliability of the information on the Internet is a major challenge to obtaining good quality information.” | Discussion with a health professional “Communicate the information to their physician. These women highlight a need to understand and interpret the meaning of their test results. They even use the Internet before going to their physician to get an explanation and interpretation of the results.” | Outcome for relationship with health professional and Health professionals’ outcomes  “Only seven women out of the fifty said that they experienced a positive reaction from their physician when they raised the issue of searching the Internet for pregnancy related information. Twelve women talked about receiving a negative reaction, when the Internet was mentioned during a visit with their physician. That reaction resulted in their avoiding the issue during subsequent visits with their doctor. The Internet is perceived by the women as something that could threaten the power status of the physician and, therefore, put the physician in an uncomfortable position.”  Outcome for health improvement “I was in a lot of stress before my amniocentesis. Because of my age, they recommended that I do the test and I feared this test, so I spent days on the Internet reading everything I could about amniocentesis. I wanted to get confirmation about the decision to do the test and read research about it to know the possible risks. I think that reading about it on the Internet made me feel more relaxed.” | 100% |
| (Liszka, Steyer, & Hueston, 2006) | Prevalence survey (quantitative descriptive): A self-administered survey questionnaire was completed by a consecutive sample of 203 patients seen at an academic family medicine faculty practice in the spring of 2004 (USA). | Ethnicity and socioeconomic status, and level of education  Bivariate analyses suggest that those who used the Internet for health information were more likely to be non-Hispanic White race and have a college education or above. | Discussion with a health professional, Use for decision-making  73 % used the online information to make a health-related decision, 50 % shared the information with their provider. | Outcome for health improvement  Overall, online health-seekers agree that the information on the Internet improved their health. | 75% |
| (Liu, C., Liu, & Xu, 2009) | Prevalence survey (quantitative descriptive): Authors reported a secondary statistical analysis of data from a national survey (N = 2,928) conducted by the Pew Internet & American Life Project (2006) on health topics (USA). | eHealth literacy  “The data suggest that active users were more likely to evaluate the credibility of online health information resources than less active users; types of users did not make a difference in searches on behalf of others.” | Discussion with a health professional, Use for decision-making  “Especially, between active and less active users, online health information had strong impacts on three specific aspects of decision-making: (1) the treatment on an illness or condition, (2) the overall approach, and (3) asking new questions.” | Outcome for health improvement or worsening  “As to the perceived usefulness, significantly more active searchers (60.5%) claimed that the information they found had major or minor impact on themselves or someone else than less active searchers (39.5%).” | 100% |
| (Liu, J. et al., 2013)* | Prevalence survey (quantitative descriptive): A cross-sectional multicenter study was conducted using a standard anonymous questionnaire that gathered demographic information and information on Internet use (N = 780) in China. | Confidence in OCHI, Access to Internet, and eHealth literacy  “We also probed the reasons for not searching for information about epilepsy on the Internet. The most common reasons reported were (1) do not know how to access or ﬁnd information on the Internet (83.2%), (2) do not trust the Internet (8.9%), (3) no access to the Internet 3.7%, and (4) other reasons 4.2%.” | Use for change of health management  “A signiﬁcant minority, 71 of 288 participants (24.7%), purchased drugs or received treatment through the Internet, and 19 had received alternative treatment after they followed advertised links on a website or communication group.” | No outcomes reported. | 75% |
| (Macias & McMillan, 2008) | Qualitative descriptive study: 31 participants aged 60 years and older who use the Internet for more than just e-mail participated in 4 focus groups in different cities (USA). | eHealth literacy  “More advanced users tended to define the Internet with positive terms that indicated it was highly accessible: available, convenient, searchable, and targeted. But for others, the Internet was a maze characterized by terms such as difficult, overwhelming, unfriendly, and confusing.” | Discussion with a health professional, Use for providing social support  “Some reported that they regularly shared health information they had found online with their doctors. Participants reported that much of their use of the Internet for health-related information occurred after a visit to the doctor. Sometimes, they might be looking up information about a friend who had been diagnosed.” | Health professionals’ outcomes and Outcome for relationship with health professional  “The interactive characteristics of the Internet allow those seniors to manage their own needs for timely information that are often in conflict with the limited time available from health care providers. Some reported that they regularly shared health information they had found online with their doctors and found this to be a positive experience, as doctors welcomed the individual’s willingness to become informed about health issues. But for many participants who had taken online information to the doctor, the experience was negative. They did not necessarily see the doctors as being averse to technology— just to the individual’s attempt to use technology without doctor’s supervision.” | 50% |
| (Mayoh, 2010) | Two-phase sequential mixed-methods research design: In phase-one, 100 participants recruited from local support groups for various chronic health conditions completed one of two questionnaires, depending on whether or not they had sought OCHI in the past. In phase-two, a descriptive phenomenological approach was adopted in order to provide rich descriptions of patients’ experiences with six participants purposefully sampled from phase-one (UK). | Integration of OCHI in encounters with professionals  “Respondents particularly noted how the patients’ approach to sharing OCHI with a health professional, and their existing relationship, had a potential effect on the perceived reaction encountered from the professionals.”  Professionals’ attitude  “Another participant noted the importance of the health professionals’ reaction to them bringing OCHI into the consultation, and expressed that they would discontinue a relationship with a health professional who reacted adversely to them bringing OCHI into the consultation.” | Discussion with a health professional, Use for providing social support  “The 44.4% (n=20) of participants who stated that they shared OCHI with others were asked with whom they were sharing. Results demonstrated that patients shared with peers with the same chronic illness, health professionals, and friends and family.” | Outcome for involvement  Participants were asked to state whether they agreed that OCHI seeking made them feel more confident about making decisions regarding their health care. Results showed that a majority (71.4%; n=30) agreed that OCHI did have this effect. “Denise [patient’s alias] stated that locating this trusted information provided her with confidence and reassurance to take a drug that she was prescribed. Claire [alias] explained that she felt able to “take a more positive view” and work in partnership with her doctor to ensure that she was getting the appropriate treatment for her condition.” | 50% |
| (Murray, E. et al., 2003) | Prevalence survey (quantitative descriptive): A telephone survey of a nationally representative sample of the American population (N = 3209) was conducted (oversampling people in poor health) in the USA. | Professionals’ attitude  “Such serious dissatisfaction was strongly related to the physician’s communication skills and perceived reaction to the information. In particular, if the physician was perceived as being threatened by the patient bringing information in, 49% of patients evinced serious dissatisfaction as defined above, compared with 11% of patients whose physician was not perceived as acting challenged.” | Discussion with a health professional, Use for decision-making  “8% had taken information from the Internet to their physician. When asked about the effect of the information in general, 97% believed that it gave patients more confidence to talk to a physician about their concerns, 96% believed that it improved patients’ understanding of their condition, and 74% said that it been beneficial to their decision-making ability.” | Outcome for relationship with health professional and Health professionals’ outcomes  “The effect of taking information to the physician on the physician-patient relationship was likely to be positive as long as the physician had adequate communication skills, and did not appear challenged by the patient bringing in information. 85% thought that it encouraged patients to follow their physician’s advice. 93% reported that having access to such information challenged physicians to be more up-to-date with the latest treatments (…). Only 6% of patients reported negative feelings, such as embarrassment as a result of taking information to their physician, but 15% had felt hurried during the consultation (…); 12% of the 256 respondents who brought information to their physician sought a second opinion from another physician, 4% changed their physician, and 1% changed their health plan.”  Outcome for satisfaction with care  “If the physician was perceived as being threatened by the patient bringing in-formation in, 49% of patients evinced serious dissatisfaction, compared with 11% of patients whose physician was not perceived as acting challenged.” | 75% |
| (Neter & Brainin, 2012) | Prevalence survey (quantitative descriptive): Based on a representative sample of the Israeli adult population, a random digital dial telephone household survey was conducted (N = 4286) in Israel. | eHealth literacy  “Overall, respondents in the high eHealth literacy group used significantly more information sources than did the low eHealth literacy group… Finally, those highly eHealth literate gained significantly more from their information search than did the low eHealth literacy group.” | Discussion with a health professional  “The benefits extended also to their interaction with the treating physician: they asked the physician significantly more questions than they would have without the digital information search, presented the physician with the information they retrieved.” | Outcome for involvement, Outcome for problem management  “They also benefited more instrumentally: the information search improved their ability to self-manage their health care needs, affected their health behaviors, and allowed them a better use of their health insurance (...) felt significantly better positioned vis-à-vis the physician than did the low eHealth literacy group.”  Organizational Outcomes  Participants recognized that OCHI may have some adverse effects, for example unnecessary visits to a physician (39%) or that it caused patients to take up more of their physician’s time (37%). | 75% |
| (Nicholas, Huntington, Williams, & Blackburn, 2001) | Prevalence survey (quantitative descriptive): 1,068 people completed an online questionnaire about information outcomes of a consumer health website in the UK. | Health status  “Signiﬁcantly, the majority of people (three out of four) did not come to the site with a particular illness or medical condition. Indeed, most (45.2%) people came to get advice about keeping ﬁt and healthy (in other words, to remain healthy) or just came out of pure curiosity (19.5%).” | Use for decision-making  “More than one in four said that Web information had resulted in a deferred visit or had actually replaced a visit to the doctor [family physician]. 84% said that they had been helped in becoming better informed; 64% being helped ‘a lot’.” | Outcome for health improvement or worsening  “47% felt that the information they found had helped in their dealings with the doctor, while just over half felt that information found had changed the way they felt about their condition. 32% of respondents went on to say that their condition had improved after having visited the site.” | 25% |
| (Pena-Purcell, 2008) | Prevalence survey (quantitative descriptive): From the original sample of 249 Hispanics and 2,477 non-Hispanic whites, a secondary data analysis was conducted using respondents who answered "yes" to the item "In the last 12 months, have you looked for any information about a health topic on the Internet?". The sample included 72 Hispanic and 883 non-Hispanic whites (USA). | Ethnicity and socioeconomic status  “Hispanics were likely to agree that Internet health information improves understanding of medical conditions and treatments, gives patients confidence to talk to doctors about health concerns.” | Discussion with a health professional  “Overall ratings were also positive for items related to sharing Internet health information with a doctor.” | Outcome for relationship with health professional and Health professionals’ outcomes  “Hispanics and non-Hispanic whites reported that physician-patient relationships worsened as a result of bringing online health information to a visit.”  Outcome for involvement  “Hispanics were less likely than non-Hispanic whites to agree that health information on the Internet improves people's understanding of medical conditions and treatments, gives patients the confidence to talk to their doctors about their concerns, and helps patients get treatment they would not get otherwise.” | 75% |
| (Pifalo, Hollander, Henderson, DeSalvo, & Gill, 1997) | Prevalence survey (quantitative descriptive.): A questionnaire was mailed to a convenience sample of 270 adults who received information from a Consumer Health Library (intervention) exploring the outcomes of information (from Internet and databases) on their decisions, actions, anxiety, and physician-patient communication (n = 239 respondents) (USA). | Age, gender, level of education, and ethnicity and socioeconomic status  “More than three-fourth of the respondents were women (77.8%, N=182). Those aged forty and over accounted for 70.9% (N=166) of the responses. Most of the respondents either were college graduates (47.0%, N=110) or had some college education (30.8%, N= 72). Eighty-eight percent of the respondents were Caucasian (N=206) and 6% were African American (N=14).” | Use for decision-making, Use for discussion with a health professional, Use for change of health management  “51.3% (N=120) had asked questions of a health care provider, and for 35.5% (N=83) a decision about treatment options was influenced, 20.1% (N=47) made a lifestyle change, 17.5% (N=41) sought a second opinion, 12.0% (N=28) made an appointment with a health care provider.” | Outcome for health improvement or worsening and Organizational outcome  “As a result of reading the information, 52.1% indicated that their anxiety about an illness or health concern was reduced, 17.5% followed instructions given by a health provider, and 9.8% indicated that their anxiety was increased.” | 75% |
| (Porter & Edirippulige, 2007) | Prevalence survey (quantitative descriptive): An online questionnaire was completed by parents of children (aged 0-21 years) with a permanent hearing loss (N = 166) living in Australia. | Level of education  Education level influences level of Internet use. Parents with a university education use the Internet more frequently for health information seeking. | Use for discussion with a health professional  “Over half the respondents (52%) had spoken to their doctor or hearing professional about the information they had found on the Internet.”  Use for decision-making “University-educated parents are more likely to ﬁnd information on the Internet which has a major inﬂuence on decisions they made about their child's management.” | No outcomes reported. | 50% |
| (Powell, Inglis, Ronnie, & Large, 2011) | Mixed methods sequential design: An online questionnaire survey was completed by NHS Direct users (National Health System website) (N = 796). The, a purposeful subsample of survey respondents (n = 26) participated in in-depth semi structured interviews by telephone or instant messaging/email (UK). | Gender  Women were more likely than men to seek help for someone else or both themselves and someone else. | Use for discussion with a health professional “Interviewees recognized that the Internet played a role in allowing them to become informed consumers, better able to share decisions with their health care provider.” | Outcome for health improvement or worsening and Organizational outcomes  “So quite often I get reassurance that I’m not an odd one out from this. An interviewee described how reassurance over a bloodshot eye eliminated the need for a general practice appointment. Misuse of accurate information, leading to inappropriate self-diagnosis [was also reported].” | 50% |
| (Rice, 2006) | Prevalence survey (quantitative descriptive): This paper provides results from seven major nationally representative datasets from the Pew Internet & American Life Project (USA). | Gender, and Health status  “A variety of outcomes from or positive assessments of searching for Internet health information are predicted most strongly by sex (female); more speciﬁc health reasons, belonging to an online support group sharing health interests, and helping another deal with an illness or major health condition.” | Use for discussion with a health professional  “Being more likely to ask one’s doctor new questions or seek a second opinion from another doctor [was linked] to those who have more health reasons for going online (4%).” | Outcome for involvement  “An improvement in the way one takes care of their health was predicted by more health reasons for going online, more frequent online health seeking, participation in an online support group, and greater perceived credibility of online health information.” | 50% |
| (Rideout, 2001) | Prevalence survey (quantitative descriptive): This reported results of a nationally representative, random dial telephone survey of 1,209 respondents aged between 15 and 24, including an over- sample of approximately 200 African American and Latino youth (USA). | Ethnicity and socioeconomic status  “African Americans who have sought health information are more likely to report changing their behavior than others, with fully half (52%) saying they have done so (42% of Hispanics and 37% of whites).”  OCHI characteristics “Confidentiality is one of the most important concerns for young people seeking health information.” | Use for decision-making  “One in seven (14%) have seen a doctor or other health provider because of health information they got online.”  Use for discussion with a health professional “Many young people who have looked up health information online say they have had conversations with friends, family members and health providers about the information they found.” | Outcome for involvement  “Four out of ten (39%) say they have changed their personal behavior because of health information they got online.” | 50% |
| (Rogers & Mead, 2004) | Qualitative descriptive study: Data were collected via (a) semi-structured interviews with (and observations of) a purposeful sample of five patients who used a free primary care-based Internet service, and (b) interviews with a purposeful sample of 12 people on patient attitudes to using the Internet for health information (UK). | Confidence in OCHI  “Those who were unfamiliar with and sceptical about this intervention were more likely to place their faith in health services which, by contrast, they viewed as being much more reliable and accessible, and open to all comers, whatever their social background.” | Use for decision-making  “For users of the Internet clinic, information was seen as a time-saving alternative to see a health professional.”  Use for discussion with a health professional  “For users of the Internet clinic, information-use enabled them to discuss more confidently with health professionals, e.g., about a referral [to a specialist].” | Outcome for health worsening  “At its worst, information was anxiety-inducing as it was seen as a source of interference both with established ways of coping and with the efforts of experts working in hard- pressed services.” | 75% |
| (Shaikh, Shaikh, Kamal, & Masood, 2008)* | Prevalence survey (quantitative descriptive): An anonymous self-administered questionnaire on Internet (access, usage patterns, health information seeking behavior, and beliefs about reliability of such information) was distributed to students enrolled in graduate programs (N = 598) in Pakistan. | Confidence in OCHI  “Out of one hundred and thirty- nine students who had used Internet for health related information, 109 (78.4%) thought that such information was reliable.” | Use for discussion with a health professional  “Out of 139 students who had used Internet for seeking health information, 35 (25.2 %) students replied affirmatively to the question of having discussed health information obtained from Internet with their physician whom they visited for any illness/treatment.” | No outcomes reported | 75% |
| (Shinchuk, Chiou, Czarnowski, & Meleger, 2010) | Prevalence survey (quantitative descriptive): Patients completed an anonymous two-page questionnaire on the day of their regularly scheduled appointment with their pain-management healthcare provider (N = 89) (USA). | Professionals’ attitude, and level of education  “Subjects who received encouragement from their healthcare providers to obtain online pain-related medical information (OPRMI) were significantly more likely to do so than those who did not. Subjects who surfed for OPRMI were more likely to have a higher level of education.” | Use for discussion with a health professional  “Only half of our subjects ever shared the information found on Internet with their treating healthcare provider.” | No outcomes reported. | 50% |
| (Siegel et al., 2006) | Non-randomized study and prevalence survey (quantitative non-randomized and descriptive): Two comprehensive evaluation studies of an information prescription program (Information Rx) were conducted between 2002 and 2005 by the American College of Physicians Foundation (ACPF) and the U.S. National Library of Medicine (NLM). Study-1 was a pre-post design. A pre-program mailed baseline questionnaire was completed in 2003 by ACPF members in Iowa and Georgia (n=489). Approximately nine months after the program was launched in both states, a mailed program evaluation questionnaire was completed by ACPF members (N = 270). Study-2 was a nationwide survey of patients and health care providers who participated in the program in 2005 (USA). | Confidence in OCHI  “74% of the patients indicated that they respond quickly to a doctor’s recommendation to try something new to improve their health care. 75 % of respondents indicated that they ﬁrst learned about MedlinePlus from their physician. 84% also stated that they were more inclined to trust the information on MedlinePlus because it was prescribed to them by their physician.” | Use for decision-making  93% of patients reported that the information helped them make better health decisions, 70% reported that the found information improved their understanding of an illness or a health condition, 45% reported that the found information may influence their future health decisions.  Use for discussion with a health professional  “56% of patients who found needed information on MedlinePlus, planned to discuss it with their doctor and 46% with family and friends.” | Outcome for health worsening  “An equal proportion of reporting physicians expressed concern that additional information could increase anxiety for some patients.” | 75% |
| (Siliquini et al., 2011) | Prevalence survey (quantitative descriptive): A multicenter survey was administered in six Italian cities. The sample included 3018 people aged 18-65 years. About 65% of respondents reported using the Internet, and 57% of them reported using it to search for health-related information (Italy). | Age and chronic disease  “A significantly higher risk of negative behaviors was found with increasing age up to 53 years (p = .05), while the absence of chronic diseases decreased the risk of negative behaviors (p = .004).” | Use for decision-making  “The most relevant and significant results were found in the “self-medication” and “negative behaviors” categories. The modifications in “choices in the provision of health” and “positive behaviors” were not correlated with e-health use.” | Outcome for health worsening  “The rate of users adopting any dangerous modifications of behavior based on information found on the Internet was quite high and noteworthy, especially in those with chronic diseases. These results are very important because such behaviors may be dangerous, especially if they are executed on the basis of false information.” | 75% |
| (Sillence, Briggs, Harris, & Fishwick, 2007) | Qualitative descriptive study: 15 women at various stages of menopause, residing in the North East of England, with different levels of education, were recruited through advertisements in local media. Data were collected on their online information seeking behaviors using observations, personal diaries and telephone interviews (UK). | eHealth literacy  “Most individuals preferred sites that were run by reputable organizations or had a medical or expert ‘feel’. They trusted the information on such websites, especially when the credentials of the site and its authors were made explicit.” | Use for decision-making, Use for confirmation, Use for discussion with a health professional  “The extract is typical in illustrating the way in which participants would use online information to prepare themselves for a discussion with friends or physicians, and also illustrates the way in which consumers were motivated to engage in particular activities and would use the Internet as a source of evidence to support their choices. The online information and advice inﬂuenced patients’ decision making without threatening their desire to communicate with physicians.” | Outcome for relationship with health professional  “Women felt that the Internet inﬂuenced their decision-making and improved communications with physicians. They also reported a new found conﬁdence with respect to doctors and medical information.” | 75% |
| (Sommerhalder, Abraham, Zufferey, Barth, & Abel, 2009) | Qualitative descriptive study: Semi-structured interviews about OCHI usage in medical consultations were conducted with 32 patients and 20 physicians in the city of Bern between December 2005 and September 2007 (Switzerland). | Integration of OCHI in encounters with professionals  “Patients used different strategies to introduce health-related Internet information during consultations and to ask for their physicians’ evaluations of the health-related Internet information.” | Use for discussion with a health professional  “Discussing patients’ concerns and answering patients’ questions were important elements of successful consultations with Internet-informed patients to achieve clarity, orientation and certainty. These patients stated that they would bring up the health-related Internet information if they had information that contradicted the physician’s interpretation of the clinical situation.” | Outcome for relationship with health professional and Health professionals’ outcomes  “Discussing health-related Internet information with patients was appreciated by most of the physicians but misleading interpretations by patients and contrary views compared to physicians caused conﬂicts during consultations. In these situations physicians tried to explain the correct interpretation of the health-related Internet information, which was described as a time-consuming procedure. Some physicians were upset by these situations. They interpreted it as a sign of lacking trust in their medical expertise.” | 75% |
| (Stern, Cotten, & Drentea, 2012) | Prevalence survey (quantitative descriptive): Data came from the 2006 Pew Internet & American Life Project’s telephone survey of people aged 18 years and older (nationwide random digit sample of telephone numbers selected from telephone exchanges in continental USA). Of the 2,928 respondents, 1,990 were Internet users and included in this study on the use and outcomes of OCHI. | Gender  “Turning to whether the information affected a health-related decision (…), parental status is not associated with putting this information into use; however, once again, sex does have a significant effect.” | Use for discussion with a health professional  Participants were more confident to ask new questions to a health care professional. | Outcome for problem management  Health information found on the Internet changed the way participants cope with a chronic condition or how they managed pain. | 50% |
| (Takahashi et al., 2011) | Prevalence survey (quantitative descriptive): A cross-sectional survey of a quasi-representative sample of the population aged 15–79 years (N = 1200) was conducted in September 2007 (Japan). | Age, ethnicity and socioeconomic status, and level of education  “We observed that participants over 50 years of age were significantly less likely to use the Internet via personal computer for acquiring health-related information, while those with an income over ¥10,000,000 [$90,000 USD] or with more than 12 years of education were more likely to acquire information this way.” | Use for change of health management  “More than two-thirds of Internet users ‘strongly agreed’ or ‘agreed’ that Internet use (…) “affected the way I eat or exercise”, while only 23% thought it “improved my ability to manage my health care needs without visiting a doctor or other health care provider”.”  Use for discussion with health professional  “Perceived effects of Internet use on health-related activities included experiences of telling health professionals about health-related information from the Internet among Internet users.” | Outcome for health worsening  “A small percentage [of respondents] reported increased anxiety after online health information.” | 50% |
| (Walsh, Hyde, Hamilton, & White, 2012) | Non-randomized cohort study (quantitative non-randomized): Parents completed a baseline questionnaire on child care (N = 391). Two months later, 187 parents completed a follow-up questionnaire assessing their decisions to use OCHI with regard to their child’s health care (Australia). | Confidence in OCHI “Parents with a more positive attitude toward using online information (who perceive greater social pressure/support to use this information) believe they have greater control and that there are lower risks associated with the behaviours. Other mothers who have similar attitudes and behaviours and have limited medical experience will have stronger intentions to use online information for child health care.” | Use for decision-making  “[…] parents with stronger intentions to use online information to diagnose/treat their child’s health issues (…) are more likely to actually do so.” | No outcomes reported. | 75% |
| (Warner & Procaccino, 2004) | Prevalence survey (quantitative descriptive): A questionnaire was completed by a convenience sample of 119 women (New Jersey and eastern Pennsylvania) on health information seeking and their awareness of speciﬁc health and medical information resources. The 10-page questionnaire was developed using several questions from validated survey instruments (USA). | Age  “Preliminary statistical evidence revealed a relation between age and the number of times the Web had been used for looking for health information, the highest frequency of usage falling generally in the 35–64 age range.” | Use for discussion with a health professional  “Eighty-two percent of respondents indicated that they had talked to a doctor, nurse or medical professional about the information they found.” | Outcome for involvement and for health improvement  “Eighty-three percent of 111 respondents indicated that information they located has affected their decisions about treatments; 80% of 104 have used the information to improve the way they eat, 65% of 103 used the information to improve the way they exercised, and 75% of 99 reported that the information affected their decisions about treatments or their care of someone else.” | 50% |
| (Weaver, Thompson, Weaver, & Hopkins, 2009) | Prevalence survey (quantitative descriptive): A self-administered online survey was conducted in summer 2006 among a sample of adults living in the Seattle-Tacoma designated market area; 562 people aged 19-90 years responded (USA). | Gender  “Female respondents (71.4%) were more than 2.5 times more likely (odds ratio, 2.56; 95% CI=1.44–4.54) to be in the non-adherent [to medical recommendations] cohort than male (28.6%) respondents.” | Use for change of health management  “The inﬂuence of Internet health information on healthcare nonadherence decisions – assessed through the question ‘‘Have you ever refused or discontinued treatment recommended by your doctor/dentist based on information you obtained from the Internet?” – was evident. Speciﬁcally, 11.2% of respondents reported Internet-instigated non-adherence.” | Organizational outcomes  “Speciﬁcally, 11.2% of respondents reported Internet-instigated non-adherence.” | 75% |
| (Ybarra & Suman, 2008) | Prevalence survey (quantitative descriptive): Data from Surveying the Digital Future Year 4, a nationally representative longitudinal telephone survey of Americans (aged 12 years and older) was used to examine the reasons for, assessments of, and actions taken as a result of OCHI (USA). | Age and eHealth literacy  “In general, assessment of the experience worsened as age increased. For example, 16% of adolescents as compared with 15% of young adults, 20% of middle-aged adults and 31% of older adults wanted more information, but did not know where to ﬁnd it. Similarly, no adolescents reported that trying to ﬁnd information took a lot of effort, as compared with 10% of young adults, 14% of middle-aged adults and 23% of older adults.”  Gender  “Once the information was obtained, men were signiﬁcantly more likely to report trying to diagnose a problem compared with women (p = 0.02), whereas women were signiﬁcantly more likely than men to seek support from others (p < 0.01).” | Use for decision-making  “Respondents are equally likely to contact a health care provider because of information found online regardless of sex or age.”  “Once the information was obtained, men were signiﬁcantly more likely to report trying to diagnose a problem compared with women (p = 0.02), whereas women were signiﬁcantly more likely than men to seek support from others (p <0.01).” | Outcome for satisfaction with care  “Regardless of age, the great majority (>70%) of health information seekers reported feeling satisﬁed with the information they found, and they most often (>70%) felt more comfortable with information received from a health provider after their online experience. (…) Findings suggest that irrespective of one’s age and sex, the online seeking experience is generally positive and reinforces the patient–provider relationship. Several important distinctions among age groups and between men and women are noted.” | 50% |
| (Yoo & Robbins, 2008) | Prevalence survey (quantitative descriptive): A self-administered survey questionnaire was completed by a purposeful sample of middle-aged women (N = 354) living in a mid-sized city in the USA. | Level of education  Confidence in OCHI. | Use for decision-making  Under “gratiﬁcations sought” from OCHI, there were two items found to be of significance in the questionnaire: “To decide about whether or not to visit a doctor” and “to decide about how to treat an illness” | No outcomes reported. | 75% |

Design: According to authors (categories of the [Mixed Methods Appraisal Tool](http://mixedmethodsappraisaltoolpublic.pbworks.com/w/page/24607821/FrontPage))

OCHI: Online Consumer Health Information

*Legend: While three studies (4.6%) were conducted outside OECD countries (China, Pakistan and Turkey), removing these studies from the synthesis did not influence the results as no outcome was uniquely reported in these studies. This can be seen as strength (qualitative theoretical generalizability) or limitation (non-homogeneity). Of 65 included studies, 62 (95.4%) were conducted in 12 OECD countries (Organisation for Economic Co-operation and Development). In OECD countries, primary care services and internet use are aligned. Health indicators and access to health services vary a little,(Davis, Stremikis, Squires, & Schoen, 2014) but almost 99% of the adult population can access the internet, which directly influences OCHI outcomes (access → OCHI → outcome). Regarding health services and internet use, the main differences between the 3 non-OECD versus the 12 OECD countries can be summarized as follows. In China, primary health care settings usually provide the traditional Chinese medicine, and family physicians are unevenly distributed (Li et al., 2017); more than half of the population use the internet (55.8% in December 2017) (China Internet Network Information Center, 2018). In Pakistan, health care services are mostly private and unevenly distributed (low access) (World Health Organization, 2017); only about one-fifth of the population has access to the internet. In Turquey, a reform of health services has recently increased access to primary care services (Hone et al., 2017), and about two-third of the population use the internet.

# Appendix 4

# Harmonization of Themes: Outcomes of Online Consumer Health Information

|  | Five levels of outcomes | Definition of OCHI outcomes | Examples from included studies |
| --- | --- | --- | --- |
| 1. | Situational relevance of OCHI | Outcome of OCHI associated with the achievement of the patient’s information needs in a specific situation. Situational relevance is a prerequisite for the other four levels of outcomes. | - In the study, the majority (64%) of participants were able to find the needed information in less than 15 minutes, and 80% were able to find the information within 30 minutes. However, 13% reported that they were unable to find the information they were looking for (Amirault et al., 2005). - An interviewee stated: “If I am searching for something on the Internet [using a search engine] and I get millions of websites and 90% of the results are not a good fit with what I am looking for, I get frustrated and leave the search” (Lev, 2009). |
| 2. | Cognitive Impact of OCHI | Outcomes of OCHI associated with a change in patient's attitude about behaviour or subjective (perceived) norm. | Examples from included studies |
| 2.1 | Impact on learning | OCHI outcome associated with a change in patient's subjective (perceived) norm resulting in acquisition of new knowledge or understanding (e.g., when a consumer says: ‘this information helped me to better understand a particular health issue’). | - The majority of study participants indicated that using the Internet for health information, improved at least some aspect of their knowledge about health care issues (Baker et al., 2003). - Among parents who searched for OCHI, 599 (81.2%) said that (a) information they found improved their understanding of health issues, and (b) they learned more about an illness or a specific symptom (Bianco et al., 2013). - On a scale of 1 (strongly agree) to 4 (strongly disagree), Hispanic participants were less likely than non-Hispanic Whites to agree that OCHI improves people's understanding of medical conditions and treatments (Pena-Purcell, 2008). |
| 2.2 | Impact on memory | OCHI outcome associated with a change in patient's subjective (perceived) norm resulting in the consumer remembering something (e.g., when a consumer says: ‘I am reminded of something I already knew’). | - No quote (term and concept derived from the initial model). |
| 2.3 | Impact on motivation to learn | OCHI outcome associated with a change in patient's attitude resulting in the patient’s desire for more information (e.g., when a consumer says: ‘Now I want to learn more about this health issue’). | - The intended effect of the Internet training program was to increase the study participants’ desire for more health information, which can be satisfied by their use of the Internet (Campbell, 2009). - Among participants, 36% reported that because of the information found on MedlinePlus, they wanted to look for more health information (Siegel et al., 2006). |
| 2.4 | Impact on satisfaction with information | OCHI outcome associated with a change in patient's attitude resulting in a positive or a negative critical evaluation of information (e.g., when a consumer says: ‘I think there is a problem with this information’). | - Several study participants commented on the cultural sensitivity of information found on the website; e.g., some requested more content in French (Burton-Jeangros & Hammer, 2013). - Some participants were concerned with information bias, questioning information providers’ ﬁnancial gains, in which case they were less likely to trust commercial health information sources (Powell et al., 2011). |
| 2.5 | Impact on safety | OCHI outcome associated with a change in patient's attitude resulting in fear that the information may cause damage or harm (e.g., when a consumer says: ‘I think this information can be harmful’). | - A few participants were concerned with the quality of online health information. They were particularly worried about potential harm associated with inconsistencies in information from different websites (Lagan et al., 2011). |
| 2.6 | Impact on worry | OCHI outcome associated with a change in patient's attitude resulting in feeling more anxious, or feeling calmer (e.g., when a consumer says: ‘Now I am reassured’). | - Consulting the Internet after a consultation had a reassuring effect on some study participants; e.g., one participant stated that he consulted the Internet “to see if what the physician tells me matches with what I find elsewhere” (Caiata-Zufferey et al., 2010). - As a result of reading the information, 52% of participants indicated that their anxiety about an illness or health concern was reduced (Pifalo et al., 1997). |
| 2.99 | Other impact | OCHI outcomes associated with a change in patient's attitude about behaviour or subjective (perceived) norm not listed in the concepts presented in 2.1 to 2.6 (e.g., when a consumer says: ‘I cannot assess the impact of this information on my knowledge’). |  |
| **3** | Use of OCHI | Outcomes associated with the patient’s intention to use or behavioural use of OCHI. | Examples from included studies |
| 3.1 | Use for decision-making | OCHI outcome associated with an intention to use information for choosing what to do (e.g., when a consumer says: ‘I did not know what to do, and this information helped or will help me make a decision about my health’). | Among study participants, 24% used the information to decide whether to visit their family physician, while another 8% indicated that they used the information they found to decide not to do the visit (Amirault et al., 2005). |
| 3.2 | Use for confirmation | OCHI outcome associated with an intention to use information to be more certain (e.g., when a consumer says: ‘I knew what to do, and I used or will use this information to be more certain about my health care’). | - A study participant stated: “Usually (75% of the time) after looking at online health information, I conclude that what the GP [family physician] has said is right and therefore I’ll take my medicine and be quiet”(Kivits, 2006). - A participant was referred for an amniocentesis, but she was stressed and worried about this test. She reported having spent “days” on the Internet reading everything she could find about amniocentesis. Reading helped her to know more about the test and feel more confident about doing it (Lev, 2009). |
| 3.3 | Use for change of health management | OCHI outcome associated with an intention to use or a behavioural use of information to modify how they manage their health (e.g., when a consumer says: ‘I was doing or going to do something concerning my health, and used or will use this information to do it differently’). | - Study participants who visited the National Health System website reported that they used the acquired information to seek further treatment and change their health care (Amirault et al., 2005). - Study results suggest the more frequently a person uses the Internet as a source of health information, the more likely they are to change their health behavior (Ayers & Kronenfeld, 2007). - OCHI helped 322 (44%) participants become more aware of their eating habits; 169 (34%) increased their physical activity, and 138 (10%) increased their participation in screening programs (Bianco et al., 2013). |
| 3.4 | Use for discussion with a health professional | OCHI outcome associated with an intention to use or a behavioural use of information in conversation with a health professional (e.g., when a consumer says: ‘I used or will use this information in discussion with a health professional’). | - About one third of study participants reported having shared the information they found online with their health care provider (Amirault et al., 2005). - Among participants, 36% had discussed information they found online on their illness and treatment with their health care provider (Anderson, 2004). |
| 3.5 | Use for providing social support | OCHI outcome associated with an intention to use or a behavioural use of information to help a family member or a friend (e.g., when a consumer says: ‘I used or will use this information to support a family member with a health condition’). | - Among study participants, 63 (81%) indicated they were going to share the health information they found with others (Amirault et al., 2005). - A majority of participants (59%) who were looking for OCHI for someone else, reported that information they found affected the way they helped care for someone else (Laflamme, 2003). |
| 3.99 | Other use | OCHI outcomes associated with an intention to use or a behavioural use of information not listed in the concepts presented in 3.1 to 3.5 (e.g., when the consumer says: ‘I still do not know if I will use this information’). |  |
| 4 | Health and health care related OCHI outcomes | A positive or a negative change in patient’s perspective on health (including health care and wellbeing) following the patient’s behavioural use of OCHI. | Examples from included studies |
| 4.1 | Outcome for satisfaction with care | OCHI outcome associated with a change in the patient’s perspective on health care, health, or wellbeing following behavioural use of information resulting in feeling pleased or not with the received care (e.g., when a consumer says: ‘This information made me more satisfied with the health care I received’). | - Study participants reported higher satisfaction and lower concern when health care providers validated their efforts and took OCHI seriously, in comparison to situations when providers disagreed with information they found online (Bylund et al., 2007). |
| 4.2 | Outcome for relationship with health professional | OCHI outcome associated with a change in the patient’s perspective on health care, health, or wellbeing following behavioural use of information resulting in an improvement or a deterioration in communication with a health professional (e.g., when a consumer says: ‘This information allowed me to better communicate with a health professional’; or says: ‘Sharing this information led to a breakdown in communication with a health professional’). | - Among study participants, only 25% discussed the information they found online with their physician; 79% believed that OCHI use did not change their relationship with their physician; 13% believed it had a positive effect, while 8% believed it had a negative effect (Bianco et al., 2013). - Study results suggest OCHI contributes to subtle changes in the relationship between patients and health professionals, rather than a dramatic transformation (Hart et al., 2004). |
| 4.3 | Outcome for involvement | OCHI outcome associated with a change in the patient’s perspective on health care, health, or wellbeing following behavioural use of information resulting in feeling more or less committed to make health decisions (e.g., when a consumer says: ‘With this information, I am more engaged in decisions about my health’). | - Three-quarters of study participants felt better prepared and able to participate in decision-making about medical treatments (Dolan et al., 2004). - Out of participants aged between 31 and 45 years, 66% reported asking more questions during office visits, 54% followed physician advice more closely, and 54% made self-directed dietary changes (Iverson et al., 2008). |
| 4.4 | Outcome for problem management | OCHI outcome associated with a change in the patient’s perspective on health care, health, or wellbeing following behavioural use of information resulting in feeling more or less able to deal with a health problem (e.g., when a consumer says: ‘This information helped me to better handle a problem with my health’). | Study participants experienced instrumental benefits: information searches improved their ability to manage their health care needs, affected their health behaviours, and facilitated better use of their health insurance (Neter & Brainin, 2012). |
| 4.5 | Outcome for prevention | OCHI outcome associated with a change in the patient’s perspective on health care, health, or wellbeing following behavioural use of information resulting in feeling more or less able to avoid a health problem (e.g., when a consumer says: ‘This information helped me to prevent a health problem or the worsening of a health problem’). | Pregnant women searched online to know what they should and should not eat during pregnancy; e.g., one participant stated: “I did not know how many cups of coffee per day I am allowed to drink when I am pregnant, so I searched the Internet to get an answer about it” (Lev, 2009). |
| 4.6 | Outcome for health improvement or worsening | OCHI outcome associated with a change in the patient’s perspective on health care, health, or wellbeing following behavioural use of information resulting in feeling more or less able to have better health and wellbeing (e.g., when a consumer says: ‘This information helped me to improve my health’). | - Some study participants connected pursuing latest health information, being healthy and achieving positive health outcomes; e.g., one participant stated: “I search the Internet; I look up a lot of health things; I’m really into being healthy and trying to find out about the latest things” (Berg, 2011). - Overall, study participants agreed that OCHI improved their health (Liszka et al., 2006). - Among participants, 25% reported feelings of anxiety after finding information on health or illness from the Internet (compared to 39% who reported feeling reassured) (Bujnowska-Fedak et al., 2007). |
| 4.99 | Other health and health care related OCHI outcomes | OCHI outcomes associated with a change in the patient’s perspective on health care, health, or wellbeing following behavioural use of information having positive or negative effects not listed in the concepts presented in 4.1 to 4.6 (e.g., when a consumer says: ‘I do not know how OCHI affects my perception of health care’). |  |
| **5** | OCHI outcomes affecting health care services | A change in health care services following the patient’s behavioural use of OCHI. | Examples from included studies |
| 5.1 | Health professionals’ outcomes | OCHI outcome associated with a change in a health care service following patient’s behavioural use of information resulting in a change of the health professional’s practice. | - Some physicians discussed strategies for “firing” the patient who use OCHI in clinical encounters, or referring these patients to specialists, or charging for extratime (Ahmad et al., 2006). - Not all physicians embrace the role of OCHI interpreter and helper; some are resistant to discussing OCHI with patients; e.g., a physician stated: “Most of them [patients] know it’s annoying to me when they do it [bring in OCHI downloads], so they don’t. (…) I just sort of stick with what I know and what I do and how I practice” (Ahmad et al., 2006). |
| 5.2 | Organizational outcomes | OCHI outcome associated with a change in a health care service following patient’s behavioural use of information resulting in an increase or a decrease in health system use (e.g., when a patient says: ‘Using information increased the number of visits to the clinic’). | - One third (36%) of study participants reported that OCHI had improved their ability to manage their health care without visiting a health care provider (Cohall et al., 2011). - Participants recognized that OCHI may have some adverse effects such as unnecessary visits to a physician (39%), or may lead patients to use more physicians’ time (37%) (Murray, E. et al., 2003). - Participants reported a variety of negative effects of OCHI such as increasing health care cost due to inappropriate health service utilisation (56%) and interfering with time efﬁciency of the visit (61%) (Kim & Kim, 2009). |
| 5.99 | Other outcomes affecting health care services | OCHI outcome associated with a change in a health care service following patient’s behavioural use of information not listed in the concepts presented in 5.1 and 5.2. |  |

OCHI: Online Consumer Health Information

Appendix 5. Harmonization of Themes: Consumers’ Online Health Information Needs Driving Their Seeking Behaviour

|  | OCHI needs driving seeking behaviour | Reasons for consumers’ searches for and acquisition of online health information | Examples from included studies (consumers including primary care patients, family caregivers and the general public) |
| --- | --- | --- | --- |
| 1 | Acquisition for personal clarification | Consumers search for online health information to answer a question about his/her own health (e.g., when a consumer says: ‘I looked for information to answer a question about my health’). | - Among study participants, 42% searched for OCHI for themselves. As a participant stated: “It’s my health! It’s normal that I want to know everything that relates to my body! I would search everywhere to better understand things that have consequences for me. I think it is a right and a duty” (Amirault et al., 2005). |
| 2 | Acquisition for clarification for others | Consumers search for online health information to answer a question about the health of a family member, a friend, a neighbour, a colleague, an employer, or a client (e.g., when a consumer says: ‘I looked for information to answer a question about the health of someone else’). | - The second most common reason behind searches for OCHI was to find health information for others such as a family member or a friend (15%) and for an employer or a client (10%) (Amirault et al., 2005). - Among study participants, 27% sought OCHI for relatives, 12 % for children, 9 % for friends and 4 % for colleagues (Harbour & Chowdhury, 2007). |
| 3 | Acquisition for curiosity | Consumers search for online health information to know more about a specific health topic or to stay up-to-date (e.g., when a consumer says: ‘I looked for information to satisfy my curiosity about a health matter’). | - Searches for OCHI can be motivated by general curiosity, and a desire to know more and keep up to date with current trends (Berg, 2011). - Among study participants, 20% searched for OCHI by curiosity (Nicholas et al., 2001). |
| 4 | Acquisition for decision-making | Consumers search for online health information to decide on a course of action such as choosing a treatment option (e.g., when a consumer says: ‘I looked for information to help me decide if I should see a health professional’). | - Among study participants aged between 15 and 30 years, 142 (30%) reported having searched for OCHI to decide if they visit their family physician, which was linked to avoiding a visit (Beck et al., 2014). - Among study participants, 27% reported that they searched for OCHI to decide whether they need a visit to their family physician, and that OCHI had replaced a visit at least once (Nicholas et al., 2001). |
| 5 | Acquisition for visit preparation | Consumers search for online health information to be ready for an encounter with a health professional (e.g., when a consumer says: ‘I looked for information to prepare myself before talking to a health professional’). | - Interviewees reported better preparation of clinical encounters with their family physician by looking for OCHI before the encounter: they either used the information they found to prepare questions in advance or a discussion. They linked this preparation to better understanding of and participation in clinical encounters (Kivits, 2006). |
| 6 | Acquisition for visit follow-up | Consumers search for online health information to add to personal knowledge (e.g., when a consumer says: ‘I looked for information to add to what I already knew’). | - Among parents who searched for OCHI, 170 (23%) reported that they used the Internet to find more information than that provided by their family physician (Bianco et al., 2013). - Four out of nine participants used the Internet to look for more information related to their family physicians’ recommendations (Berg, 2011). |
| 7 | Acquisition for health management alternatives | Consumers search for online health information to explore choices different from those given by a health professional (e.g., when a consumer says: ‘I looked for information to find different choices from those given by health professional’). | - After the consultation with their doctor, a few patients searched for alternatives to what was discussed and decided. The alternatives they searched for usually concerned treatments, e.g., medication or surgical techniques to see whether other possibilities exist with fewer side effects (Caiata-Zufferey et al., 2010). - One study participant stated: “I spent one hour on the Internet looking for news for kidney disease, healing, cure, therapy, and alternative approaches“ (Berg, 2011) |
| 8 | Acquisition for confirmation | Consumers search for online health information to reinforce personal knowledge (e.g., when a consumer says: ‘I looked for information to be more certain’). | - The initial motivations for OCHI seeking are related to the needs for increasing knowledge, decreasing uncertainty and finding alternative viewpoints (Caiata-Zufferey et al., 2010). - One study participant stated: “I was searching the Internet after each and every blood test. I had to understand what the results mean. It’s helping me to decrease my level of anxiety because I can find out if my tests results are normal” (Lev, 2009). |
| 99 | Other reasons for acquisition | Consumers search for online health information for reasons not listed in the concepts presented in 1 to 8. |  |

OCHI: Online Consumer Health Information

Appendix 6

Card Sorting Exercise: Contextual Factors of OCHI Needs and Outcomes

| 1 | Individual characteristics | Definitions (consumers’ demographic, socioeconomic and health characteristics) | Examples from the literature and included studies |
| --- | --- | --- | --- |
| 1.1 | Gender | Consumers’ self-reported gender | Information studies usually show that women are more likely to search for OCHI for themselves and others (compared to men), while findings of an included study suggest men participants were more likely discussing OCHI with health professionals (Chung, 2013). |
| 1.2 | Age | Consumers’ age | Information studies usually show that people aged 31-45 years are more likely to search for OCHI (compared to other age groups). In one included study, participants aged 31-45 years were more likely using OCHI to ask questions during clinical encounters, and make self-directed dietary changes (Iverson et al., 2008). |
| 1.3 | Level of education | Consumers’ level of education (no diploma or completed diploma) | Information studies usually show that people with a higher level of education are more likely to search for OCHI. One included study suggested participants with a lower level of education were less likely to search for and use OCHI in primary health care (Baker et al., 2003). |
| 1.4 | Ethnicity and socioeconomic status | Consumers’ self-reported ethnicity and socioeconomic status | Information studies usually show that people with a higher socioeconomic status (and knowledge of information available in the locally dominant language and culture) are more likely to search for OCHI; e.g., an included study suggests wealthier participants were more likely to search for and use OCHI (Murray, T., Hagey, Willms, Shillington, & Desjardins, 2008). |
| 1.5 | Health status | Consumers’ state of physical, mental and social well-being (e.g., complete well-being vs. physical, mental or social issue) | Study participants who reported their general health as being fair or poor sought OCHI more often than participants who reported their health as being excellent, very good or good (Bansil et al., 2006). |
| 1.6 | Chronic disease | Consumers with or without a chronic disease or multiple diseases | Study participants who reported one or more chronic diseases (e.g., depression) were more likely to search OCHI compared to participants without chronic disease (Bansil et al., 2006). |
| 1.7 | eHealth literacy | Consumers’ ability to seek, find, understand, and appraise OCHI and apply the knowledge gained to addressing health issues (concept combining knowledge, competences and skills pertaining to health information literacy and computer literacy) | - Among study participants, about one third (29%) stated they sometimes have trouble for finding good quality OCHI, and assessing the quality of OCHI (Anderson, 2004). - Few participants had information literacy and computer literacy skills; they relied on others (intermediaries) to find and appraise OCHI (Hart et al., 2004). |
| 1.8 | Confidence in OCHI | Consumers’ self-reported level of trust in OCHI (attitude) | Among study participants, 7% did not trust OCHI and some reported the lack of trust in OCHI as a reason for not using it (Dolan et al., 2004). |
| 1.99 | Other individual factors not presented in 1.1 to 1.8 |  |  |
| 2 | Social and technical factors | Definitions | Examples from included studies |
| 2.1 | Social network | Consumers with or without a supportive social network (family members and caregivers, friends, neighbours, peers and education-health-social professionals) | A study participant reported that she has a chronic disease and uses the Internet for both OCHI and obtaining social support from her network; she is a ‘tech-savvy Web user’ and easily find OCHI, and she is motivated to connect with others through the Internet because of her small social network (Berg, 2011). |
| 2.2 | Access to the Internet | Consumers with 24/7 easy access or lack of access to Internet and high speed Internet | Results of an included study suggest access to the Internet is associated with seeking, finding and using OCHI (Amirault et al., 2005). |
| 2.3 | OCHI characteristics | Characteristics of OCHI influencing how consumers find, understand and apply it | Study participants reported advantages of OCHI in terms of convenience, coverage and anonymity (compared to health information from other sources). A participant contrasted these advantages with barriers to access health care services (Powell et al., 2011). |
| 2.99 | Other social and technical factors not presented in 2.1 to 2.3 |  |  |
| 3 | Relationships with professionals | Definitions | Examples from included studies |
| 3.1 | Satisfaction with professionals’ behaviour | Consumers’ satisfaction with health professionals including information they provide during clinical encounters and prescriptions of trustworthy OCHI sources | Study results suggest patient-physician conflict may be derived from not trusting health professionals whose behavior is incompatible with OCHI, or from discrepancies between OCHI and information provided by professionals; among study participants, 86% found additional information (after a clinical encounter) about their health problem, and 70% about their treatment (Bianco et al., 2013). |
| 3.2 | Professionals’ attitude | Consumers’ perception of health professionals’ attitudes toward OCHI | Among study participants, about 10% felt that their family physician would be upset if they used OCHI, and another 16% were uncertain how their physician would respond to this (Anderson, 2004). |
| 3.3 | Integration of OCHI in encounters with professionals | Consumers’ strategies used to present and discuss, or avoid mentioning, OCHI in clinical encounters with health professionals | Study participants reported using a variety of strategies to introduce OCHI during encounters with a health professional: 56% explicitly stated that their information comes from the Internet, whereas 44% did not mention the Internet as their source of information (Bylund et al., 2007)*.* |
| 3.99 | Other factors linked to relationships with professionals (not presented in 3.1 to 3.3) |  |  |
| 4 | Education, health and social services | Definitions | Examples from included studies |
| 4.1 | Access to services | Barriers and facilitators to access education. health and social services | Study participants seek, find and apply OCHI to avoid barriers for accessing primary care services, such as difficulties in getting an appointment; e.g., a 25-year-old woman participant stated: “It takes two hours and two buses to get to the doctor (...). It’s easier to use [the Internet] than to get down to the doctor (...). It’s the time and the money, you know, those kinds of factors and then all the problems with getting appointments as well” (Powell et al., 2011). |
| 4.99 | Other education, health and social services factors |  |  |

OCHI: Online Consumer Health Information

**References (appendices)**

Ahmad, F., Hudak, P. L., Bercovitz, K., Hollenberg, E., & Levinson, W. (2006). Are physicians ready for patients with Internet-based health information? *Journal of Medical Internet Research, 8*(3), e22. doi:10.2196/jmir.8.3.e22

Amirault, M., Cobbett, S., Doherty, A., Hartigan-Rogers, J., LeBlanc, A., Muise-Davis, M., & Newell, J. (2005). Consumer health information on the Internet: an evaluation report on the Nova Scotia Health Network. *Journal of the Canadian Health Libraries Association, 26*(2), 41-46. doi:<http://dx.doi.org/10.5596/c05-013>

Anderson, J. (2004). Consumers of e-Health: Patterns of Use and Barriers. *Social Science Computer Review, 22*(2), 242-248. doi:<http://dx.doi.org/10.1177/0894439303262671>

Aronson, J., Heneghan, C., Mahtani, K., & Plüddemann, A. (2018). A word about evidence:‘rapid reviews’ or ‘restricted reviews’? *BMJ evidence-based medicine*, e111025.

Ayers, S. L., & Kronenfeld, J. J. (2007). Chronic illness and health-seeking information on the Internet. *Health: an Interdisciplinary Journal for the Social Study of Health, Illness & Medicine, 11*(3), 327-347. doi:10.1177/1363459307077547

Baker, L., Wagner, T. H., Singer, S., & Bundorf, M. K. (2003). Use of the Internet and e-mail for health care information: results from a national survey. *JAMA, 289*(18), 2400-2406. doi:10.1001/jama.289.18.2400

Bansil, P., Keenan, N. L., Zlot, A. I., & Gilliland, J. C. (2006). Health-related Information on the Web: Results From the HealthStyles Survey, 2002–2003. *Preventing Chronic Disease, 3*(2), A36.

Beck, F., Richard, J.-B., Nguyen-Thanh, V., Montagni, I., Parizot, I., & Renahy, E. (2014). Use of the internet as a health information resource among French young adults: results from a nationally representative survey. *Journal of Medical Internet Research, 16*(5), e128. doi:10.2196/jmir.2934

Berg, K. A. (2011). *Health Management in the Age of the Internet.* University of Toronto.

Bianco, A., Zucco, R., Nobile, C. G. A., Pileggi, C., & Pavia, M. (2013). Parents seeking health-related information on the Internet: cross-sectional study. *Journal of Medical Internet Research, 15*(9), e204. doi:10.2196/jmir.2752

Booth, A., Noyes, J., Flemming, K., Gerhardus, A., Wahlster, P., van der Wilt, G., . . . Rehfuess, E. (2016). *Guidance on choosing qualitative evidence synthesis methods for use in health technology assessments of complex interventions*. Retrieved from London:

Bujnowska-Fedak, M. M., Staniszewski, A., & Steciwko, A. (2007). A survey of Internet use for health purposes in Poland. *Journal of Telemedicine and Telecare, 13*(suppl 1), 16-19. doi:<https://doi.org/10.1258/135763307781645130>

Burton-Jeangros, C., & Hammer, R. (2013). [Information seeking on the internet: what information are pregnant women seeking?]. *Revue Medicale Suisse, 9*(383), 895-897.

Bylund, C. L., Gueguen, J. A., Sabee, C. M., Imes, R. S., Li, Y., & Sanford, A. A. (2007). Provider-patient dialogue about Internet health information: an exploration of strategies to improve the provider-patient relationship. *Patient Education & Counseling, 66*(3), 346-352. doi:10.1016/j.pec.2007.01.009

Caiata-Zufferey, M., Abraham, A., Sommerhalder, K., & Schulz, P. J. (2010). Online health information seeking in the context of the medical consultation in Switzerland. *Qualitative Health Research, 20*(8), 1050-1061. doi:<http://dx.doi.org/10.1177/1049732310368404>

Campbell, R. (2009). Internet-based health information seeking among low-income, minority seniors living in urban residential centers. *Home Health Care Management & Practice, 21*(3), 195-202. doi:10.1177/1084822308322648

Carroll, C., Booth, A., Leaviss, J., & Rick, J. (2013). “Best fit” framework synthesis: refining the method. *BMC Medical Research Methodology, 13*(1), e37. doi:10.1186/1471-2288-13-37

Chalmers, I., Hedges, L., & Cooper, H. (2002). A brief history of research synthesis. *Evaluation and the Health Professions, 25*(1), 12-37. doi:10.1177/0163278702025001003

China Internet Network Information Center. (2018). *Statistical report on Internet development in China*  Retrieved from <https://cnnic.com.cn/IDR/ReportDownloads/201807/P020180711391069195909.pdf>

Chung, J. E. (2013). Patient-provider discussion of online health information: results from the 2007 Health Information National Trends Survey (HINTS). *Journal of Health Communication, 18*(6), 627-648. doi:<http://dx.doi.org/10.1080/10810730.2012.743628>

Coberly, E., Boren, S. A., Davis, J. W., McConnell, A. L., Chitima-Matsiga, R., Ge, B., . . . Hodge, R. H. (2010). Linking clinic patients to Internet-based, condition-specific information prescriptions. *Journal of the Medical Library Association, 98*(2), 160-164. doi:10.3163/1536-5050.98.2.009

Cohall, A. T., Nye, A., Moon-Howard, J., Kukafka, R., Dye, B., Vaughan, R. D., & Northridge, M. E. (2011). Computer use, internet access, and online health searching among Harlem adults. *American Journal of Health Promotion, 25*(5), 325-333. doi:<http://dx.doi.org/10.4278/ajhp.090325-QUAN-121>

Couper, M. P., Singer, E., Levin, C. A., Fowler, F. J., Jr., Fagerlin, A., & Zikmund-Fisher, B. J. (2010). Use of the Internet and ratings of information sources for medical decisions: results from the DECISIONS survey. *Medical Decision Making, 30*(5 Suppl), 106S-114S. doi:<http://dx.doi.org/10.1177/0272989X10377661>

Creswell, J., & Plano Clark, V. (2018). *Designing and conducting mixed methods research* (3rd ed.). Thousand Oaks: SAGE.

Davis, K., Stremikis, K., Squires, D., & Schoen, C. (2014). *Mirror, mirror on the wall: How the performance of the US Health care system compares internationally*. Retrieved from New York: <https://www.commonwealthfund.org/sites/default/files/documents/___media_files_publications_fund_report_2014_jun_1755_davis_mirror_mirror_2014.pdf>

Diaz, J. A., Griffith, R. A., Ng, J. J., Reinert, S. E., Friedmann, P. D., & Moulton, A. W. (2002). Patients' use of the Internet for medical information. *Journal of General Internal Medicine, 17*(3), 180-185. doi:10.1046/j.1525-1497.2002.10603.x

Dixon-Woods, M., Cavers, D., Agarwal, S., Annandale, E., Arthur, A., Harvey, J., . . . Sutton, A. (2006). Conducting a critical interpretive synthesis of the literature on access to healthcare by vulnerable groups. *BMC Medical Research Methodology, 6*(35), 1-13. doi:10.1186/1471-2288-6-35

Dolan, G., Iredale, R., Williams, R., & Ameen, J. (2004). Consumer use of the internet for health information: a survey of primary care patients. *International Journal of Consumer Studies, 28*(2), 147-153. doi:10.1111/j.1470-6431.2003.00363.x

Ettel, G., 3rd, Nathanson, I., Ettel, D., Wilson, C., & Meola, P. (2012). How do adolescents access health information? And do they ask their physicians? *Permanente Journal, 16*(1), 35-38.

Fox, S., Rainie, L., & Horrigan, J. (2006). The Online Health Care Revolution: How the Web Helps Americans Take Better Care of Themselves. Washington, DC: Pew Internet & American Life Project; November 2000. *PIP_Health_Report. pdf*.

Gauld, R., & Williams, S. (2009). Use of the Internet for health information: a study of Australians and New Zealanders. *Informatics for Health and Social Care, 34*(3), 149-158. doi:<http://dx.doi.org/10.1080/17538150903102448>

Gough, D., Thomas, J., & Oliver, S. (2012). Clarifying differences between review designs and methods. *Systematic Reviews, 1*(28), 1-9. doi:10.1186/2046-4053-1-28

Grant, M. J., & Booth, A. (2009). A typology of reviews: an analysis of 14 review types and associated methodologies. *Health Information & Libraries Journal, 26*(2), 91-108. doi:10.1111/j.1471-1842.2009.00848.x

Greenhalgh, T., Robert, G., Macfarlane, F., Bate, P., Kyriakidou, O., & Peacock, R. (2005). Storylines of research in diffusion of innovation: A meta-narrative approach to systematic review. *Social Science & Medicine, 61*(2), 417-430. doi:10.1016/j.socscimed.2004.12.001

Harbour, J., & Chowdhury, G. G. (2007). Use and outcome of online health information services: a study among Scottish population. *Journal of Documentation, 63*(2), 229-242. doi:<https://doi.org/10.1108/00220410710737196>

Hardey, M. (2001). "E-health": the Internet and the transformation of patients into consumers and producers of health knowledge. *Information, Communication & Society, 4*(3), 388-405.

Hart, A., Henwood, F., & Wyatt, S. (2004). The role of the Internet in patient-practitioner relationships: findings from a qualitative research study. *Journal of Medical Internet Research, 6*(3). doi:10.2196/jmir.6.3.e36

Heyvaert, M., Hannes, K., & Onghena, P. (2016). *Using mixed methods research synthesis for literature reviews* (1st ed. Vol. 4). Los Angeles: SAGE.

Higgins, J., & Green, S. (2011). Cochrane Handbook for Systematic Reviews of Interventions Version 5.1.0 [updated March 2011]. Retrieved from <http://handbook.cochrane.org>

Hone, T., Gurol-Urganci, I., Millett, C., Başara, B., Akdağ, R., & Atun, R. (2017). Effect of primary health care reforms in Turkey on health service utilization and user satisfaction. *Health policy and planning, 32*(1), 57-67.

Hong, Q., Fabregues, S., Bartlett, G., Boardman, F., Cargo, M., Dagenais, P., . . . Pluye, P. (In Press). The Mixed Methods Appraisal Tool (MMAT) version 2018 for information professionals and researchers. *Education for Information, Special Issue*.

Hong, Q., & Pluye, P. (In Press). Systematic reviews: A brief historical overview. *Education for Information, Special Issue*.

Hong, Q., Pluye, P., Bujold, M., & Wassef, M. (2017). Convergent and sequential synthesis designs: implications for conducting and reporting systematic reviews of qualitative and quantitative evidence. *Systematic Reviews, 6*(1), e61. doi:10.1186/s13643-017-0454-2

Hong, T. (2008). Internet health information in the patient-provider dialogue. *Cyberpsychology & Behavior, 11*(5), 587-589. doi:<http://dx.doi.org/10.1089/cpb.2007.0172>

Houston, T. K., & Allison, J. J. (2002). Users of Internet health information: differences by health status. *Journal of Medical Internet Research, 4*(2), e7. doi:10.2196/jmir.4.2.e7

Iverson, S. A., Howard, K. B., & Penney, B. K. (2008). Impact of internet use on health-related behaviors and the patient-physician relationship: a survey-based study and review. *Journal of the American Osteopathic Association, 108*(12), 699.

Jensen, L. A., & Allen, M. N. (1996). Meta-synthesis of qualitative findings. *Qualitative Health Research, 6*(4), 553-560. doi:10.1177/104973239600600407

Kavathe, R. (2009). *Patterns of access and use of online health information among Internet users: A case study.* Bowling Green State University.

Kavlak, O., Atan, Ş. Ü., Güleç, D., Öztürk, R., & Atay, N. (2012). Pregnant women's use of the internet in relation to their pregnancy in Izmir, Turkey. *Informatics for Health and Social Care, 37*(4), 253-263. doi:10.3109/17538157.2012.710686

Khechine, H., Pascot, D., & Premont, P. (2008). Use of health-related information from the Internet by English-speaking patients. *Health Informatics Journal, 14*(1), 17-28. doi:<http://dx.doi.org/10.1177/1460458207086331>

Kim, J., & Kim, S. (2009). Physicians' perception of the effects of internet health information on the doctor–patient relationship. *Informatics for Health and Social Care, 34*(3), 136-148. doi:10.1080/17538150903102422

Kivits, J. (2006). Informed patients and the internet: a mediated context for consultations with health professionals. *Journal of Health Psychology, 11*(2), 269-282. doi:10.1177/1359105306061186

Laflamme, D. J. (2003). Online health information-seeking, health locus of control and health literacy among low-income internet users in east baltimore. *Dissertation Abstracts International: Section B: The Sciences and Engineering, 64*(2-B), 666.

Lagan, B. M., Sinclair, M., & Kernohan, W. G. (2011). What is the impact of the Internet on decision-making in pregnancy? A global study. *Birth, 38*(4), 336-345. doi:10.1111/j.1523-536X.2011.00488.x

Lev, E. (2009). *Googling While Expecting: Internet Use by Israeli Women during Pregnancy.* Ohio University.

Li, X., Jiapeng, L., Shuang, H., Cheng, K., De Maeseneer, J., Meng, Q., & al., e. (2017). The primary health-care system in China. *The Lancet, 390*(10112), 2584 - 2594.

Liszka, H. A., Steyer, T. E., & Hueston, W. J. (2006). Virtual medical care: how are our patients using online health information? *Journal of Community Health, 31*(5), 368-378.

Littell, J. (2018). *Conceptual and practical classification of research reviews and other evidence synthesis products*. Retrieved from Oslo:

Liu, C., Liu, Y. H., & Xu, T. (2009). To search is to believe? A comparative study of health information use by internet users. *Proceedings of the American Society for Information Science & Technology, 46*(1), 1-5.

Liu, J., Liu, Z., Zhang, Z., Dong, S., Zhen, Z., Man, L., & Xu, R. (2013). Internet usage for health information by patients with epilepsy in China. *Seizure, 22*(9), 787-790. doi:<http://dx.doi.org/10.1016/j.seizure.2013.06.007>

Macias, W., & McMillan, S. (2008). The return of the house call: the role of internet-based interactivity in bringing health information home to older adults. *Health Communication, 23*(1), 34-44. doi:<http://dx.doi.org/10.1080/10410230701805174>

Mayoh, J. (2010). *"It gave me relief... it gave me confidence" - The online health information seeking experiences of adults with chronic health conditions.* Bournemouth University.

Murray, E., Lo, B., Pollack, L., Donelan, K., Catania, J., White, M., . . . Turner, R. (2003). The impact of health information on the internet on the physician-patient relationship: patient perceptions. *Archives of Internal Medicine, 163*(14), 1727-1734. doi:10.1001/archinte.163.14.1727

Murray, T., Hagey, J., Willms, D., Shillington, R., & Desjardins, R. (2008). *Health literacy in Canada: a healthy understanding 2008*. Ottawa, ON: Canadian Council on Learning.

Neter, E., & Brainin, E. (2012). eHealth literacy: Extending the digital divide to the realm of health information. *Journal of Medical Internet Research, 14*(1), 359-368. doi:<http://dx.doi.org/10.2196/jmir.1619>

Neuendorf, K. A. (2002). *The content analysis guidebook*. Thousand Oaks, CA: SAGE Publications.

Nicholas, D., Huntington, P., Williams, P., & Blackburn, P. (2001). Digital health information provision and health outcomes. *Journal of Information Science, 27*(4), 265-276. doi:<https://doi.org/10.1177/016555150102700409>

Noblit, G. W., & Hare, R. D. (1988). *Meta-ethnography: Synthesizing qualitative studies* (Vol. 11). Thousand Oaks, CA: SAGE Publications.

Pawson, R., Greenhalgh, T., Harvey, G., & Walshe, K. (2005). Realist review - A new method of systematic review designed for complex policy interventions. *Journal of Health Services Research & Policy, 10* (Suppl 1), 21-34. doi:10.1258/1355819054308530

Pena-Purcell, N. (2008). Hispanics' use of Internet health information: an exploratory study. *Journal of the Medical Library Association, 96*(2), 101-107. doi:<http://dx.doi.org/10.3163/1536-5050.96.2.101>

Petticrew, M. (2001). Systematic reviews from astronomy to zoology: Myths and misconceptions. *British Medical Journal, 322*(7278), 98.

Petticrew, M., Rehfuess, E., Noyes, J., Higgins, J. P., Mayhew, A., Pantoja, T., . . . Sowden, A. (2013). Synthesizing evidence on complex interventions: How meta-analytical, qualitative, and mixed-method approaches can contribute. *Journal of Clinical Epidemiology, 66*(11), 1230-1243. doi:10.1016/j.jclinepi.2013.06.005

Pifalo, V., Hollander, S., Henderson, C. L., DeSalvo, P., & Gill, G. P. (1997). The impact of consumer health information provided by libraries: the Delaware experience. *Bulletin of the Medical Library Association, 85*(1), 16.

Pluye, P., Hong, Q., Bush, P., & Vedel, I. (2016). Opening-up the definition of systematic literature review: the plurality of worldviews, methodologies and methods for reviews and syntheses. *Journal of Clinical Epidemiology, 73*, 2-5. doi:10.1016/j.jclinepi.2015.08.033

Pluye, P., Hong, Q., Granikov, V., & Vedel, I. (In Press). The wiki toolkit for planning, conducting and reporting mixed studies reviews. *Education for Information, Special Issue*.

Pluye, P., & Hong, Q. N. (2014). Combining the power of stories and the power of numbers: mixed methods research and mixed studies reviews. *Public Health, 35*(1), 29. doi:10.1146/annurev-publhealth-032013-182440

Popay, J., Roberts, H., Sowden, A., Petticrew, M., Arai, L., Britten, N., . . . Duffy, S. (2006). *Guidance on the conduct of narrative synthesis in systematic reviews: Final report*. Swindon: ESRC Methods Programme.

Pope, C., Mays, N., & Popay, J. (2007). Synthesizing qualitative and quantitative health research. Berkshire, UK: Open University Press.

Porter, A., & Edirippulige, S. (2007). Parents of deaf children seeking hearing loss-related information on the Internet: The Australian experience. *Journal of Deaf Studies and Deaf Education, 12*(4), 518-529. doi:<http://dx.doi.org/10.1093/deafed/enm009>

Powell, J., Inglis, N., Ronnie, J., & Large, S. (2011). The characteristics and motivations of online health information seekers: cross-sectional survey and qualitative interview study. *Journal of Medical Internet Research, 13*(1). doi:10.2196/jmir.1600

Randolph, J. J. (2009). A guide to writing the dissertation literature review. *Practical Assessment, Research & Evaluation, 14*(13), 2.

Rice, R. E. (2006). Influences, usage, and outcomes of Internet health information searching: multivariate results from the Pew surveys. *International Journal of Medical Informatics, 75*(1), 8-28. doi:10.1016/j.ijmedinf.2005.07.032

Rideout, V. (2001). How young people use the Internet for health information. *Public Health Reports, 116*(6), 627-627.

Rogers, A., & Mead, N. (2004). More than technology and access: primary care patients' views on the use and non-use of health information in the Internet age. *Health & Social Care in the Community, 12*(2), 102-110. doi:10.1111/j.0966-0410.2004.00473.x

Sandelowski, M., Docherty, S., & Emden, C. (1997). Focus on qualitative methods - Qualitative metasynthesis: Issues and techniques. *Research in Nursing and Health, 20*, 365-372.

Shaikh, I. A., Shaikh, M. A., Kamal, A., & Masood, S. (2008). Internet access and utilization for health information among university students in Islamabad. *Journal of Ayub Medical College, Abbottabad: JAMC, 20*(4), 153-156.

Shinchuk, L. M., Chiou, P., Czarnowski, V., & Meleger, A. L. (2010). Demographics and attitudes of chronic-pain patients who seek online pain-related medical information: implications for healthcare providers. *American Journal of Physical Medicine & Rehabilitation, 89*(2), 141-146. doi:10.1097/PHM.0b013e3181c56938

Siegel, E. R., Logan, R. A., Harnsberger, R. L., Cravedi, K., Krause, J. A., Lyon, B., . . . Lindberg, D. A. (2006). Information Rx: evaluation of a new informatics tool for physicians, patients, and libraries. *Information Services & Use, 26*(1), 1-10.

Siliquini, R., Ceruti, M., Lovato, E., Bert, F., Bruno, S., De Vito, E., . . . La Torre, G. (2011). Surfing the internet for health information: an italian survey on use and population choices. *BMC Medical Informatics & Decision Making, 11*, 21. doi:<http://dx.doi.org/10.1186/1472-6947-11-21>

Sillence, E., Briggs, P., Harris, P. R., & Fishwick, L. (2007). How do patients evaluate and make use of online health information? *Social Science & Medicine, 64*(9), 1853-1862. doi:10.1016/j.socscimed.2007.01.012

Sommerhalder, K., Abraham, A., Zufferey, M. C., Barth, J., & Abel, T. (2009). Internet information and medical consultations: experiences from patients’ and physicians’ perspectives. *Patient Education and Counseling, 77*(2), 266-271. doi:10.1016/j.pec.2009.03.028

Stern, M. J., Cotten, S. R., & Drentea, P. (2012). The Separate Spheres of Online Health: Gender, Parenting, and Online Health Information Searching in the Information Age. *Journal of Family Issues, 33*(10), 1324-1350. doi:10.1177/0192513X11425459

Takahashi, Y., Ohura, T., Ishizaki, T., Okamoto, S., Miki, K., Naito, M., . . . Nakayama, T. (2011). Internet Use for Health-Related Information via Personal Computers and Cell Phones in Japan: A Cross-Sectional Population-Based Survey. *Journal of Medical Internet Research, 13*(4). doi:<http://dx.doi.org/10.2196/jmir.1796>

Tricco, A. C., Langlois, E. V., & Straus, S. E. (2017). *Rapid reviews to strengthen health policy and systems: A practical guide*. Geneva: World Health Organization.

Veinot, T. C., Senteio, C. R., Hanauer, D., & Lowery, J. C. (2017). Comprehensive process model of clinical information interaction in primary care: results of a “best-fit” framework synthesis. *Journal of the American Medical Informatics Association, 25*(6), 746-758.

Walsh, A. M., Hyde, M. K., Hamilton, K., & White, K. M. (2012). Predictive modelling: parents' decision making to use online child health information to increase their understanding and/or diagnose or treat their child's health. *BMC Medical Informatics & Decision Making, 12*, 144. doi:<http://dx.doi.org/10.1186/1472-6947-12-144>

Warner, D., & Procaccino, J. D. (2004). Toward wellness: women seeking health information. *Journal of the American Society for Information Science & Technology, 55*(8), 709-730. doi:10.1002/asi.20016

Weaver, J. B., Thompson, N. J., Weaver, S. S., & Hopkins, G. L. (2009). Healthcare non-adherence decisions and internet health information. *Computers in Human Behavior, 25*(6), 1373-1380. doi:<https://doi.org/10.1016/j.chb.2009.05.011>

Whittemore, R., Chao, A., Jang, M., Minges, K. E., & Park, C. (2014). Methods for knowledge synthesis: An overview. *Heart & Lung, 43*(5), 453-461. doi:10.1016/j.hrtlng.2014.05.014

Wong, G., Greenhalgh, T., Westhorp, G., Buckingham, J., & Pawson, R. (2013a). RAMESES publication standards: meta-narrative reviews. *BMC Med, 11*, 20. doi:10.1186/1741-7015-11-20

Wong, G., Greenhalgh, T., Westhorp, G., Buckingham, J., & Pawson, R. (2013b). RAMESES publication standards: realist syntheses. *BMC Med, 11*, 21. doi:10.1186/1741-7015-11-21

World Health Organization. (2017). *Primary health care systems (PRIMASYS): Case study from Pakistan (Licence: CC BY-NC-SA 3.0 IGO)*. Retrieved from Geneva: <http://www.who.int/alliance-hpsr/projects/alliancehpsr_pakistanprimasys.pdf>

Ybarra, M., & Suman, M. (2008). Reasons, assessments and actions taken: sex and age differences in uses of Internet health information. *Health Education Research, 23*(3), 512-521. doi:10.1093/her/cyl062

Yoo, E., & Robbins, L. S. (2008). Understanding middle-aged women's health information seeking on the Web: a theoretical approach [corrected] [published erratum appears in J AM SOC INF SCI TECHNOL 2008 May;59(7):1191]. *Journal of the American Society for Information Science & Technology, 59*(4), 577-590.

Zhao, S. (1991). Metatheory, metamethod, meta-data-analysis: What, why, and how? *Sociological Perspectives, 34*(3), 377-390.
